# Active discovery of organic semiconductors

**DOI:** 10.1038/s41467-021-22611-4

**Published:** 2021-04-23

**Authors:** Christian Kunkel, Johannes T. Margraf, Ke Chen, Harald Oberhofer, Karsten Reuter

**Affiliations:** 1 Chair for Theoretical Chemistry and Catalysis Research Center, Technische Universität München, Garching, Germany; 2 Fritz-Haber-Institut der Max-Planck-Gesellschaft, Berlin, Germany

**Keywords:** Computational chemistry, Organic molecules in materials science

## Abstract

The versatility of organic molecules generates a rich design space for organic semiconductors (OSCs) considered for electronics applications. Offering unparalleled promise for materials discovery, the vastness of this design space also dictates efficient search strategies. Here, we present an active machine learning (AML) approach that explores an unlimited search space through consecutive application of molecular morphing operations. Evaluating the suitability of OSC candidates on the basis of charge injection and mobility descriptors, the approach successively queries predictive-quality first-principles calculations to build a refining surrogate model. The AML approach is optimized in a truncated test space, providing deep methodological insight by visualizing it as a chemical space network. Significantly outperforming a conventional computational funnel, the optimized AML approach rapidly identifies well-known and hitherto unknown molecular OSC candidates with superior charge conduction properties. Most importantly, it constantly finds further candidates with highest efficiency while continuing its exploration of the endless design space.

## Abstract (web summary)

Existing methods for organic semiconductor computational screening are limited by the computational demand of the process, leading to the identification of non-optimal material candidates. Here, the authors report machine learning method to guide the discovery of organic semiconductors.

## Introduction

The sheer vastness of chemical spaces^1^ has long motivated prior-to-synthesis virtual discovery. In corresponding work, promising candidate molecules or materials for refined study are often searched and identified on the basis of a small number of quantities that are deemed representative for the targeted application^2–4^. Prevalent for first-principles computational screening approaches is to calculate such descriptors at predictive quality through electronic structure theory for every candidate in a somehow enumerated chemical space or otherwise given database. Initially performed for small focused libraries, the screening is now extended to search spaces of ever increasing size and—since discovery is limited to the explicitly considered molecules or materials—to ever more systematic and exhaustive enumerations within these spaces.

Unfortunately, the combinatorial explosion characteristic for chemical versatility quickly leads to intractable numbers of candidates for such exhaustive first-principles screenings, even if based on computationally comparably undemanding descriptors. A common strategy to tackle this problem is a computational funnel^5^. Here, the exhaustive screening is only performed for computationally least-demanding descriptors or even less demanding estimates thereof. Subsequently, the large candidate set is narrowed in staged filtering and the calculation of other descriptors is only performed for smaller and smaller subsets which appear promising in terms of the previously calculated descriptors. Unfortunately, chemical diversity suggests the multi-objective (descriptor) landscape spanned over the search space to be quite rugged^6^, with molecular or materials sub-classes likely constituting separate funnels and related analogs leading to multiple local minima. This raises concerns whether the true optimum candidates can reliably be identified through such computational funneling.

An ever more appealing alternative is therefore to completely abandon the original idea to exhaustively screen a once defined chemical space or database. Instead, the explicit first-principles computation of the descriptors is restricted to candidates emerging in an iteratively refining search^7–9^. In the context of data science, this is afforded by several learning concepts, which additionally allow to even avoid predefining or a priori enumerating the search space itself. Examples include (semi-)supervised learning, meta-, transfer-, or few-shot learning and generative models^10,11^. For drug-discovery tasks^12,13^, such concepts have already been successfully employed to further accelerate molecular de novo design^14^ and drive autonomous discovery^15^. For materials discovery based on first-principles descriptors, in particular active machine learning (AML)^16^ has been explored as a most data-efficient method^17–22^.

In AML, the acquired knowledge in form of explicitly calculated descriptors is used to successively establish a surrogate model of larger and larger regions of the rugged descriptor landscape. In an iterative procedure, the predictive-quality calculations for new candidates can then also be balanced between exploitation and exploration. In exploitation, the global insight provided by the current surrogate model is used for a targeted identification of new promising candidates. In exploration, descriptors for new candidates are specifically calculated to refine and extend the surrogate model. For this, we here employ Gaussian Process Regression (GPR) and use high values of its inherent Bayesian uncertainty estimate to flag candidates (or regions in chemical space) for which an explicit descriptor calculation will maximally contribute new information.

We pursue this concept for the efficient virtual discovery of organic semiconductors (OSCs) for electronic applications. Used in organic field effect transistors (OFETs),^23^ photovoltaics (OPVs),^24^ or light emitting diodes (OLEDs),^25^ OSCs offer great versatility and novel materials’ properties, paired with a low ecologic and economic footprint. Typical OSC-constituting molecules are, however, of considerable size (e.g., 22 or 42 non-hydrogen atoms in the classic examples pentacene or rubrene, respectively) and the spanned electronic property landscapes are known to be highly sensitive even to small molecular substitutions.^26–28^ A vast number of ~10^33^ similar-sized molecules is estimated to be synthesizable^1^, raising the suspicion that presently known well-performing OSC molecular materials are not even the tip of the iceberg. This has motivated a number of preceding exhaustive screening or virtual discovery studies in more or less restricted closed subspaces.^3,5,29–34^.

In this work we first analyze a diverse set of OSC molecules to derive clear molecular-construction rules that allow to generate an in principle unlimited OSC chemical space. This space is then successively explored by the AML discovery strategy, rapidly identifying molecular candidates that are superior to well-known OSC materials in terms of their molecular electronic descriptors assessing efficient charge injection and charge mobility. Deep methodological insight is gained by analyzing and visualizing the AML exploration inside a chemical space network (CSN) containing only a subset of the design space, limited to allow its full enumeration. Even inside this truncated chemical space the AML-discovery clearly outperforms a conventional funnel approach.

## Results

### Morphing based generation of an unlimited OSC search space

**Fig. 1:**
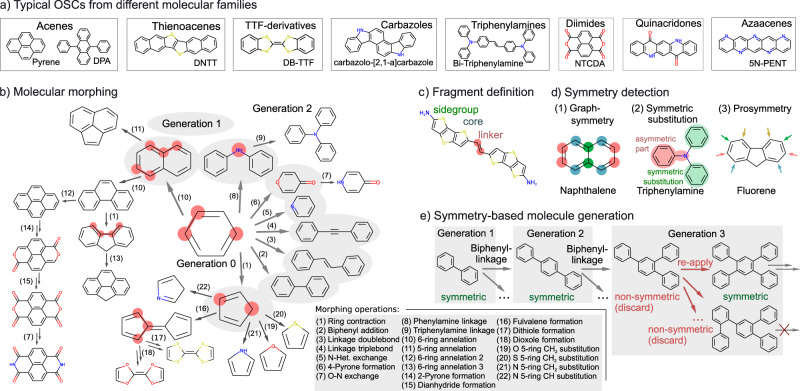
Molecular construction approach to generate an unlimited OSC chemical space. **a** Important π-conjugated molecular families and examples of well-performing OSC-molecules therein. Molecular morphing operations are designed such that the generated OSC space includes these families. **b** Schematic overview of the molecular generation process. Starting from benzene, diverse molecules are created by iterative application of up to 22 morphing operations. The first generation resulting from the 8 morphing operations applicable to benzene is fully shown. Molecules in further generations are only shown as examples, but every operation type is depicted at least once, see also Supplementary Fig. 2 for an extended depiction. **c** Fragment-definitions used throughout the text exemplified for the molecule BDTTE. Connected aromatic ring structures are cores. Linkers and sidegroups both branch from a core structure with a single bond, but are either connecting to at least two core structures or only bonded to one core fragment. **d** Concepts for symmetry detection used throughout the molecular generation process. **e** Modified molecular morphing step, adapted to the symmetry constraints imposed on candidate molecules.

The basis for our efficient AML exploration of an a priori unlimited molecular search space is the development of a concise set of molecular construction rules that allow to generate this space by iterative application. To establish a diverse, but problem-specific chemical space, we resort to existing domain knowledge and analyze the building blocks and motives contained in molecules constituting a number of well-performing crystalline OSC molecular materials. For this analysis, we exploit the fact that most functionalized organic molecules can be unambiguously fragmented into a molecular backbone (of one or more cores), linkers (that connect cores) and side groups (attached to cores) as illustrated in Fig. 1. Without loss of generality, we correspondingly fragment 30 prominent π-conjugated molecules that belong to a variety of important molecular families^23^ (Acenes, Thienoacenes, TTF-derivatives, Carbazoles, Triphenylamines, Diimides, Quinacridones and Azaacenes) and consist of the most common organic elements C, H, N, O and S. Figure 1 highlights some of these peer molecules and the full set is given in the SI in Supplementary Fig. 1. Intriguingly, the richness of chemical building blocks identified in this way can be exhaustively generated by a set of only 22 simple molecular morphing operations starting from the smallest aromatic building block benzene. As illustrated in Fig. 1 these morphing operations each act on a molecule’s individual atomic sites or fragments, each time adding, modifying or removing fragments. These morphing operations should be seen as alchemical transformations to navigate between molecules, while applying organic synthesis steps could be a viable alternative.^35^ Even though at a first glance rather unintuitive for the generation of successively larger or complex molecules, we also note that the inclusion of every morphing operation in a backwards step, i.e., resubstituting a fragment substructure, is crucial to increase the interconnectivity of the forming chemical space, see Supplementary Fig. 3.

The generic nature of the morphing operations identified through the fragmentation ansatz is not only a stepping stone for the efficient AML exploration. It also provides a blueprint for future variations of the present search space or the generation of different search spaces for other applications. Additional morphing operations will lead to more general search spaces and could be automatically extracted from a diverse chemical database^36^, while deliberate suppression of morphing operations can be used to focus on molecular sub-classes. Ring-annelation type morphing operations as well as biphenylic addition are for example essential for the iterative construction of core Acene fragments, such as in Pyrene or DPA. To build structures like Thienoacenes, Azaacenes or Carbazoles, ring contractions that lead to 5-membered rings are included as intermediates for heteroaromatic ring construction. This, though, comes at the cost of potentially yielding pericyclically reactive molecules, as discussed further below. Similarly, two types of linker operations are included to access the family of Triphenylamines. Further examples together with a detailed description of every morphing operation are provided in Supplementary Note 1. Considering their known OSC tuning potential,^28,37,38^ we note that in particular the augmentation of the present backbone-oriented set of construction rules by specific morphing operations for side groups or additional functional groups is expected to lead to an important extension of the here showcased search space.

The construction rules may also be modified to incorporate further prior knowledge about the OSC design problem. Here, we notably include constraints on molecular symmetry. Molecular symmetry may be beneficial for synthetic accessibility. Furthermore, it can mitigate mobility reducing charge localization^27^ and in particular in monomolecular crystals often favors charge percolation pathways^3,39,40^ (albeit its role can be intricate^41^). We correspondingly prune the construction rules for the present OSC context to enforce 2D graph symmetries expected to provide a prosymmetry for the 3D case. Specifically, generated molecules are only considered for further morphing, if they fall into three types of symmetry classes as explained in Fig. 1d, e: They (1) exhibit a full graph-symmetry, with all atomic environments appearing at least twice. (2) An asymmetric part in the molecule made of one or more fragments is symmetrically substituted by an even number of similar fragments, or (3) a molecule is prosymmetric such that it has atomic sites on which a single substitution operation could lead to a molecule of class (1) or (2). Further details on symmetry detection are provided in Supplementary Note 2. As always, incorporation of any such domain-specific heuristics like symmetry is thereby a double-edged sword, possibly generating more meaningful search spaces as much as introducing a limiting bias. AML is particularly appealing in this respect. Any such rules can readily be added or dropped without incurring excessive computational costs as in exhaustive screenings of predefined search spaces.

### Charge-conduction based fitness

In the spanned search space, we assess the suitability of candidate molecules for OSC applications by two descriptors known to probe two important and complementary aspects related to the conduction of charge. One concerns the efficient injection of charge from a contacting electrode into the OSC material. The other assesses the required high charge mobility inside the OSC bulk. For predominantly *p*-type OSC materials^23^ a detrimentally high barrier for a corresponding hole injection from a standard gold electrode is readily probed by a level-alignment descriptor *ϵ*_align_ = ∣*ϵ*_HOMO_ − Φ_Au_∣,^42^ which evaluates the energetic mismatch between the Au work function Φ_Au_ = − 5.1 eV^43^ and the energetic position of the highest occupied molecular orbital (HOMO) *ϵ*_HOMO_ as a common approximation of the material’s ionization potential.^44,45^ Adapting this descriptor to other electrode materials or to *n*-type OSC materials (then involving the energetic position of the lowest unoccupied molecular orbital, LUMO) is straightforward. As an equally established descriptor for the bulk charge mobility we employ the intra-molecular (hole) reorganization energy *λ*_h_, which measures the cost of accommodating a new charge state after the carrier has moved to the next molecular site.^46,47^ As molecular properties, both *ϵ*_HOMO_ and *λ*_h_ can be determined by efficient first-principles calculations as detailed in Supplementary Note 2, where the density-functional theory (DFT) B3LYP^48–50^ level of theory constitutes a well established accuracy standard^27,31,39,40,51^, matching experimental data^44,52^. We emphasize though that using the lowest-energy gas-phase conformer for the descriptor calculation disregards packing-effects in the molecular crystal^53–55^ and we further discuss the influence of conformers on descriptor values in Supplementary Note 3.

To evaluate molecular fitness and prioritize candidates during AML discovery, both objectives are combined in a scalarized fitness function

1 $$F\ =\ -\Bigg\| {\left(\begin{array}{l}{\lambda }_{{\rm{h}}}\\ {\epsilon }_{{\rm{align}}}\end{array}\right)\cdot {\bf{w}} \Bigg\| }_{2}\quad ,$$

which an ideal candidate molecule will maximize.^56^ Here, the weight vector **w** = (1.0, 0.7)^⊤^ accommodates the generally different absolute scales of the two descriptors, with the value of 0.7 chosen to yield an essentially Ohmic alignment with the electrode of ∣*ϵ*_align_∣ < 0.3 eV if *λ*_h_ falls into the range of commonly known OSCs. We note, though, that the exact choice of weights is rather unimportant for the performance of the AML search, as it only linearly biases *F* towards either of the descriptors, as further detailed below. With the currently chosen weight and at the DFT-B3LYP level of theory, pentacene and rubrene – materials that have been contacted by gold electrodes before^57,58^ – will feature *F* values of −0.16 and −0.2, respectively. A threshold *F* ≥ −0.2 will therefore later on be used to measure discovery success of the AML.

### AML: design and search strategy

By successively querying the explicit first-principles calculation of the descriptors for identified candidate molecules, the AML algorithm establishes an ever improving surrogate model of the fitness function *F* over the search space. Out of a manifold of in principle possible surrogate models, we found GPR to already achieve outstanding performance at very moderate amounts of data. In brief, the employed model uses circular Morgan fingerprints^59^ to compare the structural similarity of not yet explicitly calculated molecules with the hitherto acquired ones. Specifically, counts of substructures that can be extracted by moving up to two bonds away from each central atom are generated. The similarity between two molecules is then measured with a substructure count kernel. A full account of the GPR learning through log-marginal likelihood maximization is provided in Supplementary Note 2. A central advantage of GPR for the AML context is that it not only provides a prediction for the targeted fitness function *F*, but also the corresponding predictive uncertainty *σ* from the Gaussian variance. Balancing between exploitation and exploration, the AML algorithm can thus query new candidate molecules either because they are highly promising in terms of a maximum predicted fitness *F* or because they exhibit a high uncertainty *σ* such that their explicit calculation will maximally improve the surrogate model. Practically, molecules are thereby chosen according to an upper confidence bound acquisition function

2 $${F}_{{\rm{acq}}}=F\ +\ \kappa \sigma .$$

This represents a simple, well-tested strategy in Bayesian optimization^60–62^ or active-search^63,64^ with GPRs, which contains only one hyperparameter *κ* to balance exploration and exploitation.

Multiple possibilities arise how to actually execute the iterative AML process. After initializing the surrogate model by training on a defined number *N*_initial_ of molecules, central questions concern the acquisition of new data before the surrogate model is retrained. Compatible with super-computing resources that encourage a parallel first-principles evaluation of the descriptors for multiple molecules, we opt for a batch-based learning where *N*_batch_ molecules with maximum *F*_acq_ are queried and the model is then retrained on the basis of the accumulated new descriptor data. Future improvements could include an additional enforcement of diversity in the prioritized batch.^18,21,65,66^ In an in principle infinite chemical space, another central AML design choice regards the extent over which new molecules are practically assessed with the established, conceptually global surrogate model. Aiming for high-performance OSC molecules of tractable size and complexity, we here opt for a single tree expansion that limits the candidates to those in the vicinity of already sampled ones^67^.

In a most straightforward realization and if all molecules for which first-principles descriptors have already been computed define the current population at step *n* of the AML search, then the *N*_batch_ molecules for the next step *n* + 1 are identified in the search space formed by all molecules that can be generated by one-time application of any of the morphing operations to every molecule in the current population. While this nicely exploits the evolutionary pressure contained in the current population of size *N*_pop_ = *N*_initial_ + *n* × *N*_batch_, the search space for step *n* + 1 could also be systematically increased by exhaustive multiple-time application of the morphing operations. As illustrated below by comparing a corresponding search depth of one- or two-time application, this may help to overcome local funnels and navigate more efficiently through chemical space. On the other hand and regardless of the actual search depth *d*_search_, the continuously growing population size will at later learning steps *n* inevitably lead to a combinatorial explosion of new candidates for any such exhaustive enumeration. Eventually, this requires to decrease the resolution in the ever increasing search space. Note that precisely this combinatorial explosion also precludes popular supervised machine learning approaches that exhaustively learn molecular properties in a closed chemical space, possibly followed by some form of data mining^3^.

A decreasing resolution in the AML search space can for instance be achieved by imposing additional heuristic selection criteria, e.g., selectively suppressing certain morphing operations for increasing search depths, or other more sophisticated tree-search policies^68^ also employed in reinforcement learning^35,69^. Here, we realize deeper partial expansions of the search tree up to a search depth *d*_search_ by applying the molecular morphing operations only to a fixed number of *N*_deep_ molecules selected first from the current population and then subsequently from those molecules that were created by the previous morphing operations. By each time selecting the *N*_deep_ molecules through fitness-rank based roulette-wheel selection, i.e., by assigning higher selection probabilities to molecules with high *F*_acq_ values, the search tree is thus preferentially expanded into regions of the OSC space that the surrogate model anticipates to be rewarding (either in terms of exploitation or exploration).

### Hyperparameter optimization

The thus defined AML approach contains a number of hyperparameters that may critically affect its performance. Most notably, these are *κ* that balances exploration and exploitation in the acquisition function, *N*_batch_ the size of the prioritized batch in each learning step, as well as *d*_search_ the depth of the search space in terms of the number of applied morphing operations. The decreased resolution strategy additionally requires the specification of the fixed subset size of *N*_deep_ molecules to which morphing operations are applied. Less decisive is the initial number of molecules *N*_initial_ used for the first training of the surrogate model, which defines only an insignifiant part of the total executed first-principles calculations and which should only be large enough to somehow kick-start the AML process. Here, we suitably set *N*_initial_ to the 179 unique molecules that result in the first two generations when applying all morphing operations up to two times starting from the simplest building block benzene, cf. Fig. 1.

**Fig. 2:**
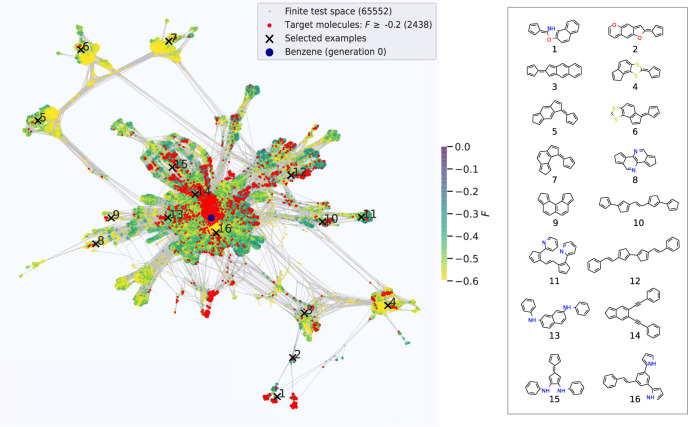
Finite OSC test space. Left panel: Chemical space network (CSN) representation of the finite OSC test space of 65.552 unique molecules generated by exhaustive application of all morphing operations up to 14 times. Each molecule is surrounded by morphing-related analogs (see text). Benzene as the smallest base molecule is colored in blue. All other molecular nodes are colored according to their fitness function *F* as calculated at the semi-empirical density-functional tight-binding level. 2438 red nodes form the target discovery group of top-performing molecules with high fitness *F* ≥ −0.2. Right panel: Example molecules from the top-performing group, chosen randomly from different areas of the CSN to illustrate the structural diversity contained in the test space.

In order to explore the effect of the other hyperparameters and optimize them for first-principles OSC discovery, we consider the finite subspace formed of all molecules up to a maximum size of 4 rings, 4 heteroatoms and 2 linkers that are generated by exhaustive application of all morphing operations up to 14 times, see Supplementary Note 2. With 65.552 unique molecules this subspace is already representative for the design problem and contains many and diversely structured high-performing molecules as illustrated in Fig. 2. At the same time, the still tractable size of the finite test space allows for the exhaustive calculation of all molecular descriptors with van der Waals (vdW) corrected density functional tight-binding (DFTB).^70^ While this semi-empirical level of theory is not fully quantitative, it provides a sufficiently realistic account of the descriptor landscape for the intended method testing as analyzed in detail in Supplementary Fig. 4. Further details on molecular test space generation and descriptor calculation are provided in the Supplementary Note 2.

The finite test space contains a total of 2438 top-performing molecules with a high fitness *F* ≥ −0.2. As a quantitative benchmark, we thus measure the discovery success *S*(*N*) as the fraction of these molecules that are identified after the descriptors of *N* molecules have been queried. With 179 queries used for the initialization, see above, the final measure *S*(5179) thus evaluates the discovery success after *n* = 50 learning steps when using *N*_batch_ = 100. Supplementary Fig. 6 compiles the corresponding success curves *S*(*N*), when systematically combining *N*_batch_ = 50, 100, or 200 with *κ* values in half-integer steps between 0 and 5, as well as for a search depth of one- or two-time exhaustive application of all morphing operations. Fortunately, we find the AML search to be highly robust with respect to the choice of *N*_batch_ and *κ*. Only a small variation of 0.71 < *S*(*N* = 5179) < 0.80 is obtained over all tested combinations for a search depth of one, meaning that 70–80% of the top-performing molecules are consistently found after descriptors for less than 8% of the entire test space have actually been computed. For a search depth of two, this success rate becomes slightly higher, reaching up to 85% as compiled in Supplementary Fig. 7. Generally, larger batch sizes seem to implicitly increase the explorative behavior, such that an almost indistinguishably optimum performance is obtained for larger *N*_batch_ in combination with successively smaller exploration weights *κ* in the acquisition function, cf. Eq. (2). For too small *κ*, the success curves become stepped though, indicating that temporarily the mainly exploitative algorithm then only meanders through identified sub-pockets of the test space. Too large *κ*, on the other hand, diminish the initial success of a then too explorative algorithm in the first learning steps. Overall, an intermediate value pair (*N*_batch_, *κ*) = (100, 2.5) thus provides a robust setting and is henceforth employed in all AML runs. For these values of (*N*_batch_, *κ*), we also performed a sensitivity analysis with regard to the employed weight vector **w** in Eq. (1) and the bond radius in the Morgan fingerprints used to assess molecular similarity. The results are summarized in Supplementary Figs. 8 and 9, respectively, and again demonstrate a high robustness with respect to these parameters.

The higher success rate for *d*_search_ = 2 indicates that it is generally advantageous to further expand the search space away from the known topologies of the current population. Assessing the dependence of the decreased resolution AML algorithm on its two additional hyperparameters, Supplementary Table 1 summarizes the corresponding discovery successes when systematically combining a varying subset size *N*_deep_ = 100, 250, 500 and 1000 with search depths *d*_search_ = 1, 2, 3, 4, 5 and 10. Again, we find the algorithm to be quite robust, with higher *d*_search_ compensating smaller *N*_deep_. Within the finite test space, many combinations thus saturate at success rates around 82–83%. This is essentially as good as the best performance of the previous exhaustive enumerations, but comes at the advantage of a controlled growth of the search space at later learning steps. For the first-principles AML discovery in the virtually unlimited OSC space below we correspondingly employ this decreased resolution search strategy with a top-performing hyperparameter combination (*d*_search_*, N*_deep_) = (3, 500).

### Visualizing AML at work

The finite test space can also be viewed as a chemical space network (CSN), in which the morphing operations establish a total of 315.451 directed connections between the constituting molecules. This allows us to visualize the space in form of a 2D graph structure, in which the molecules are mutually repelling nodes, while morphing relationships between them lead to attractive edges^71^, see Supplementary Note 1 for details. In such a representation each molecule is thus spatially surrounded by morphing-related analogs. Figure 2 shows the resulting graph, in which the individual nodes are colored according to their DFTB calculated fitness. As expected, the target group for discovery in form of the 2438 top-performing molecules is widely scattered over disjoint parts of chemical space, with ensembles of related molecules often clustered in sub-pockets.

**Fig. 3:**
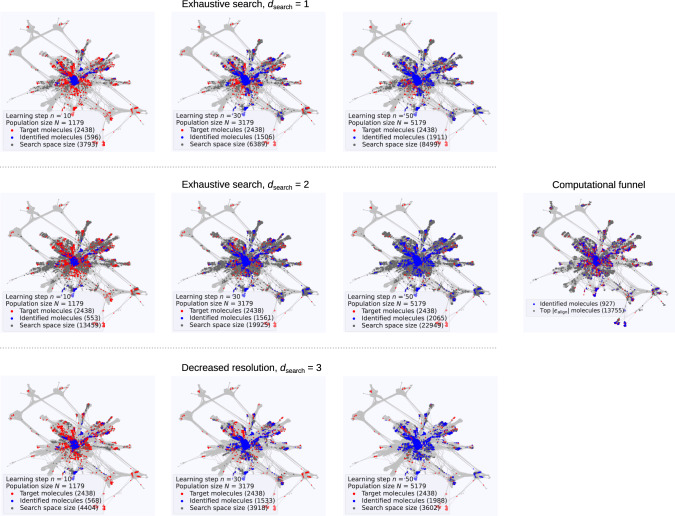
AML exploration of the finite test space. The same CSN representation of the OSC test space as in Fig. 2 is shown in gray. Superimposed are the target group of 2438 top-performing molecules in red. Each panel shows the discovery success after *n* learning steps with the color of all identified top-performing molecules changed to blue and the search space for the next learning step *n* + 1 colored in dark gray. Left upper panels: Steps *n* = 10, 30, 50 for an exhaustive search with search depth of one. Left middle panels: Steps *n* = 10, 30, 50 for an exhaustive search with search depth of two. Left lower panels: Steps *n* = 10, 30, 50 for a decreased resolution search (*N*_deep_ = 500) with search depth of three (see text). Supplementary Movies 1–3 provide the detailed, full trajectory of all three AML discovery runs over learning steps 1–50. Right centered panel: Discovery success of a conventional computational funnel after computing an equal number of descriptors (5179) as after 50 learning steps, and anticipating that knowledge of 13.755 molecules with optimum ∣*ϵ*_align_∣ < 0.3 eV is present (see text).

Apart from providing a bird’s eye view of the design problem, the CSN representation also affords a direct visual access to the AML process. Plotting the evolving population *N* over subsequent learning steps *n* reveals how much a chosen AML strategy is able to focus its exploration onto the interesting regions of chemical space and how efficiently it prioritizes OSC molecules with desired properties. Figure 3 illustrates this for the determined optimum hyperparameters and contrasts the learning for exhaustive searches with depths of one or two, with the decreased resolution strategy where the searches partially expand subsets of *N*_deep_ = 500 molecules at search depth three. For the exhaustive search with *d*_search_ = 1, the discovery is centered to more morphing-related top-performing molecules all more or less located in the core region of the CSN. In contrast, for the deeper exhaustive search, the algorithm also successfully identifies top-performing molecules in the periphery of the network that are topologically quite disconnected from the initial population. The downside is a rapidly increasing size of the search space that in the present case is only bounded by the finiteness of the considered test space. This is largely mitigated by the decreased resolution search, which nevertheless equally successfully identifies top-performing molecules at the CSN periphery.

To put this performance of the AML searches into perspective, we also contrast them in Fig. 3 with the result of a conventional computational funnel. For the latter we pretend that the calculation of *ϵ*_HOMO_ has a negligible computational cost and the value of this descriptor is known for every molecule in the test space. This allows to identify a subset of 13.755 promising molecules for which ∣*ϵ*_align_∣ < 0.3 eV and which contains all previously considered 2438 top-performing molecules. The computational funnel approach would then focus the explicit calculation of the more demanding *λ*_h_ descriptor to molecules in this subset. To enable a direct comparison with the preceding AML assessment, a random selection of 5179 molecules out of this subset would then lead to a success rate of *S*(5179) ≈ 0.4. Even in this finite test space, where the AML algorithm can not even unfold its real strength, less than half of the top-performing molecules are thus found by this prevalent computational screening strategy after spending the same amount of CPU time (assuming that the exhaustive calculation of 65.552 *ϵ*_HOMO_ descriptors for the entire test space would constitute an insignificant computational effort).

### First-principles AML discovery in a virtually unlimited OSC chemical space

Based on the gathered methodological understanding and optimized algorithmic settings (*N*_batch_ = 100, *κ* = 2.5, *d*_search_ = 3, *N*_deep_ = 500) we now proceed to first-principles AML discovery at the vdW-corrected DFT-B3LYP level of theory. This is a truly challenging endeavor, considering the vastness of the OSC design space. While the space of molecules that can be generated through the morphing operations is in principle unbounded, we here restrict it to the realm of “small molecules” containing a maximum of 100 atoms (including H atoms). This realm appears as a first, more practical target for synthesis and crystallization, also considering that essentially all known top-performing OSC molecules to date fall into this size range. Estimated to surpass a size of 10^30^ molecules, see Supplementary Note 2, the corresponding chemical space is nevertheless virtually unlimited for all practical purposes and would defy any conventional exhaustive computational screening. While an iterative search as with AML is thus the only tractable means to explore this space at predictive quality, an additional technical aspect emerges that did not yet play a role in the analysis of the finite test space at the semi-empirical level before. It concerns the typically massively parallel processing on the required high-performance computing (HPC) infrastructure. As a result of queuing or down-times, as well as convergence behavior of the first-principles calculations, the results for the *N*_batch_ descriptor calculations can become available at quite different times (or in rare cases of failed convergence or system instabilities may not become available at all). A practical way to avoid long waiting times before the last calculations are ready is to initially select a larger batch size for descriptor calculation and then continue with the forthcoming learning steps whenever the desired number of *N*_batch_ molecules has been processed (successfully or unsuccessfully). We found this strategy to afford an efficient and continuous HPC workflow, here initially submitting the 200 molecules with highest *F*_acq_ values for descriptor calculations. These are continuously processed on the HPC system by 40–100 parallel worker processes, to reach the targeted batch size *N*_batch_ = 100, while for a retraining of the surrogate model only successfully processed cases are included. In this respect, the above determined robustness of the AML performance with regard to the exact batch size also constitutes an important asset for such HPC operation.

**Fig. 4:**
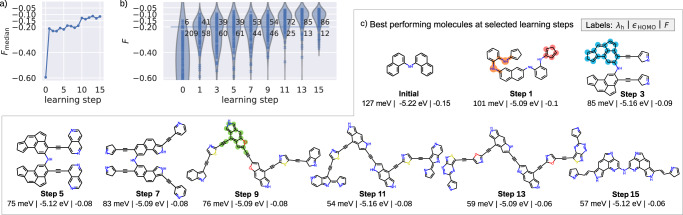
First-principles AML discovery in a virtually unlimited space. **a** Median values of molecular fitness *F* over the prioritized *N*_batch_ molecules at the different learning steps (step 0 shows the median of the initial population *N*_initial_). **b** Corresponding violin plot showing the (kernel-density estimated) distribution of molecular fitness *F* over the batch. These smooth kernel-density estimated distributions can slightly extend beyond the true range of *F* values as indicated by the explicit values marked by blue crosses. The number of queries leading to favorable and unfavorable molecules is indicated next to each violin. Due to descriptor calculation failures (see text) these numbers do not always add up to *N*_batch_ = 100. **c** Examples of top-performing molecules identified at various learning steps (see text for an explanation of the different color-highlighted geometric motifs). An extended list of the 4 top-performing molecules of each learning step is shown in Supplementary Fig. 10.

Figure 4 summarizes the results of the AML discovery run over its first 15 learning steps. Gratifyingly, the algorithm quickly stabilizes into a highly efficient mode of operation while simultaneously meandering deep into unknown chemical space. Already after five learning steps even the median fitness of the entire prioritized batch exceeds the threshold value *F* ≥ −0.2 for the first time, reflecting top-performing molecules. However, as clearly seen from the violin plots of the *F* distribution over the batches in Fig. 4b, this high efficiency does not simply result from the algorithm just exploiting its established knowledge. Even at later learning steps, the algorithm steadily queries quite unfavorable molecules with a fitness worse than *F* < −0.3. While such exploratory queries can either be based on high model uncertainty or induced by model prediction errors, they serve to continuously improve the surrogate model also outside the already considered search space. As a result, at each later learning step, the algorithm keeps on identifying top-performing molecules at a stable, high rate.

After 15 learning steps and a corresponding calculation of first-principles descriptors for 1680 molecules (and only 35 unsuccessfully terminated calculations), a total of 900 molecules with molecular fitness *F* ≥ −0.2 have been found. A relative success rate of 54%, i.e., essentially every second first-principles calculation yields a promising molecule and this without any a priori knowledge of the vast OSC space. A second AML discovery run described in Supplementary Note 4 confirms the robustness of this high performance. Notably, due to the random nature in our search strategy, significantly different, but equally favorable molecules are identified in this run. This performance becomes even more impressive from the viewpoint that these molecules are true discoveries, as essentially none of them are contained in existing focused libraries assembled in previous screening studies^3,31–34^. With typically ~10^5^ − 10^6^ entries, these data sets reflect the wealth of our existing knowledge and synthesis efforts, but simply do not even scratch the surface of the true OSC design possibilities. To this end, the negligible overlap with the top-performing molecules identified in these previous studies also has to do with molecular size. Within the first learning steps, the average size in the prioritized batch quickly rises to around 90 atoms, which is at the edge of the limit currently imposed on our search and in a size regime that could barely be addressed by the previous exhaustive enumeration studies. At the same time, even archetypical and acclaimed molecular OSC materials like DNTT (C_22_H_12_S) or rubrene (C_42_H_28_) approach this size regime, with many other experimentally tested candidates falling right into it^23^. The preferred prioritization of such larger molecules is thereby to some extent likely simply a result of the combinatorially exploding phase space. On the other hand, another physical factor could be that the AML algorithm learns and exploits the tendency of *λ*_h_ to decrease with increasing molecular size^3^ as a consequence of a larger hole delocalization (which even at the hybrid DFT-B3LYP level of theory may be slightly overestimated^72^). The inclusion of molecular coupling-sensitive descriptors into the fitness function is therefore certainly a promising topic for future studies.

The discovered molecules exhibit a diverse set of structures, incorporating distinct core fragments and the full set of allowed heteroatoms and linkers. Figure 4c illustrates this with the best-performing molecules identified at selected learning steps, and an extended list being compiled in Supplementary Fig. 11. This diversity indicates that the AML algorithm successfully explored topologically widely differing areas of the OSC space and did not get stuck in one or a few subpockets. Nevertheless, some commonalities can be spotted, like the recurrent presence of phenylamine linker motifs (marked in orange in the best-performing molecule of learning step 1 in Fig. 4c). Similarly, more complex ring systems emerged at later learning stages (marked in blue and green in the most favorable molecule of step 3 and 9, respectively) and are from thereon quite pronounced among well-performing molecules. While a diverse molecular space is searched, the AML discovery thus automatically identifies and prioritizes privileged design motifs. After harvesting a larger number of molecules in further learning steps, an exciting prospect for future studies is therefore to mine the accumulating data set and systematically extract this implicit knowledge for rational design. To this end, the trained surrogate model can also be used to quickly assess the suitability of such manually constructed molecules or of deliberate modifications of the here identified ones. The latter could be particularly appealing in view of long-term device-stability or synthetic accessibility. We note that certainly not all identified molecules are suitable in this regard. For instance, the 5-membered unsaturated rings of the displayed compound of learning step 1 (marked in red) in Fig. 4c could be problematic as they might undergo Diels-Alder type reactions, and we attribute the appearance of such ring motives as the algorithm’s intent to provide intermediates on the way to the later explored, more stable 5-membered heterocycles. Nonetheless, multiple of the favorable molecules are symmetric and composed of standard building blocks that should be easily accessible through short and reliable synthesis routes, with the surrogate model furthermore available to gauge the effect of stabilizing modifications.

## Discussion

In our view, active machine learning based on first-principles descriptors constitutes a most promising route to prior-to-synthesis virtual discovery. Its iterative refinement allows to most efficiently focus the data-generating calculations and meaningfully explore the vastness of chemical spaces at predictive quality and without a priori specifications, enumeration or reliance on empirical descriptors with limited validity range. In this work we have established such an AML discovery approach for molecular OSC materials through versatile molecular morphing operations and based on charge injection and conduction querying descriptors. Fortunately and with a view on explainable ML models, our systematic assessment within a finite test space suggests the approach to be quite robust with respect to the algorithmic hyperparameters. Most promising to further increase its already high efficiency and prevent an over-exploitation of particular structural motifs, is likely to additionally enforce structural diversity among the *N*_batch_ molecules selected at each learning step, instead of the present purely fitness-ranked roulette-wheel selection.

Central to assess this performance and enable an unbiased and systematic comparability of different AML approaches will be the establishment of well-designed, balanced and freely available benchmark platforms for unlimited search spaces. As clear from the present work, already within the here pursued single-tree expansion there are multiple design strategies and concomitant algorithmic parameters. While we have explored these in a truncated test space, AML only unfolds its full potential in the exploration of unlimited spaces. Representative and standardized benchmark platforms as already available for drug-design tasks^13^ will therefore be pivotal to truly compare various learning concepts that work without a priori enumeration or pre-definition of the search problem.

Further challenges and advancements in the physico-chemical domain comprise the adaption and extension of the molecular morphing operations to tailor the OSC search space. The present set derived from literature domain knowledge spans a design space geared towards flexible, π-conjugated molecules. Ultimately, a generic, but chemically-valid creation of morphing operations could drive discovery of many novel structural motives. Heavier requirements on the surrogate GPR-model in such cases could then be tackled with improved covariance functions for 2D molecular graphs^73^ or conformer-specific 3D coordinates^74^, while alleviating the limited scaling by sparse approximations^75^, or application of alternative models^76–79^.

Another major area for development concerns the first-principles descriptors entering the employed multi-objective fitness function. Devising such suitable descriptors has evolved into an important research area of its own^80–83^, independent of the present AML and OSC context. With the presently employed level-alignment descriptor *ϵ*_align_ and the hole reorganization energy *λ*_h_ our search readily identified a diverse range of hitherto unknown molecular candidates. Just as in conventional computational screening, there are numerous possibilities to refine the underlying candidate evaluation through additional (or alternative) descriptors. In the exemplified OSC context, obvious avenues could be to explicitly consider synthetic accessibility^84^, electronic coupling and charge-transport networks in the molecular solid^46,51,85,86^ or electron-phonon coupling^87^. In view of the high data efficiency of the AML approach, one may also drop the present focus on computationally least-demanding descriptors, originally dictated by the excessive queries in conventional exhaustive screening work. More elaborate descriptors like structural interfacing with electrode materials^88^ could therefore routinely (or at least occasionally) be requested. Eventually, one could even think of incorporating experimental feedback from self-driving laboratories^89^. The prospects are thus as manifold as exciting. Regardless of the specific road chosen, it is conceptually clear that autonomously operating workflows like the present AML approach offer an unparalleled means to accelerate the discovery and design of viable future materials like the high-mobility organic semiconductors featured in this work.

## Supplementary information

Supplementary Information

Description of Additional Supplementary Files

Supplementary Movie 1

Supplementary Movie 2

Supplementary Movie 3

## Data Availability

The source data necessary to reproduce the main figures of the manuscript is provided in the supplementary materials of this article. Source data are provided with this paper.

## Source data

Source Data

## Code availability

The code used to run AML discovery is available at 10.5281/zenodo.4554331
