# Supplementary material for: Active discovery of organic semiconductors
Source: Nat Commun. 2021 Apr 23;12:2422. doi: 10.1038/s41467-021-22611-4 (PMC8065160; doi:10.1038/s41467-021-22611-4)
Supplement: Supplementary file 1 — Supplementary Information [file 41467_2021_22611_MOESM1_ESM.pdf]

Supplementary information for Active Discovery of Organic Semiconductors

Christian Kunkel,[1] Johannes T. Margraf,[1] Ke Chen,[1] Harald Oberhofer,[1] and Karsten Reuter[1,2,a)]
[1)] *Chair for Theoretical Chemistry and Catalysis Research Center, Technische Universität München, Lichtenbergstraße 4, D-85747 Garching, Germany*
[2)] *Fritz-Haber-Institut der Max-Planck-Gesellschaft, Faradayweg 4-6, D-14195 Berlin, Germany*

Supplementary Note 1: Molecular morphing

Morphing operations are encoded as "Reaction SMARTS" that describe the changes in connectivity between a product and reactant molecule. In detail, when applying a morphing operation to a molecular graph, substructures that could be modified according to the rule are first identified. If found, the encoded modification is carried out. If the substructure could be identified multiple times, all possible outcomes are enumerated. Note, that for every morphing operation, a similar reverse operation is included in our set, carrying out the backwards transformation.

All morphing operations were derived by the fragmentation of 30 well-known OSCs shown in Supplementary Figure 1 (excluding sidegroups). A full list of all employed morphing operations is given in Supplementary Figure 2, their effects can be summarized as follows

- Ring annelation (10) allows for the construction of simple core structures such as linear Acenes (Naphthalene to Pentacene or Picene). Biphenylic addition (2) can then be used to build structures like Rubrene or Diphenylanthracene (DPA).
- Morphing operations (3),(4),(8) add linkers to an aromatic $C_{ar} - H$, leading to C_{ar}−C=C−Ph, C_{ar}−C≡C−Ph, C_{ar}−NH−Ph (Ph = Phenyl). The former linkers are included in pMSB, BDTTE or BPEA. Operation (8) was introduced as an intermediate that can be further morphed to triphenylamine moeities by (9).
- Larger annelated ring systems (i.e. Corannulene, Pyrene, Perylene, Coronene) can be derived from additional, more tailored annelation rules (11), (12), (13).
- Exchanging carbon atoms in of 6- and 5-membered rings for N, O or S, using operations (5),(20),(19),(21),(22) opens routes to Thienoacenes (DNTT, BDTT, BDTTE), carbazoles or Azaacenes (N-PENT structures). 5-membered heteroaromatic rings can thereby be derived by first contracting 6-membered rings (1).
- Tailored operations are included for the formation of diimides (NTCDI, PTCDI), dianhydrides (NTCDA, PTCDA). An exemplaric pathway is shown in Figure 1 in the main text for NTCDI. This process could start from pyrene, introducing 2-pyrones by applying (14) twice. NTCDA can be derived by applying (15) twice and repeated replacement of O by N is possible through (7), leading to NTCDI.
- Formation of Quinacridone-like structures follows a similar route but forming 4-pyrones using (6) and then (7), shown as simple pathway in Figure 1 of the main text.
- Formation of structures like DB-TTF is also possible by a separate rule. First a Fulvalene structure can be formed from single 5-membered ring CH_2 using rule (16). From thereon, applying (17) twice allows to build the core TTF structure. By analogy we decided to include (18), from which the tetraoxo derivative could be built.
- For all morphing operations, a corresponding reverse operation is included, cf. section .

While these morphing operations overall tend to give reasonable results, invalid molecules due to erroneous chemical bonding encoded in the molecular graph, can occur. The main source of these errors are the more general substructure definitions encoded in the operations. Among morphing operations that tend to produce these errors are (1) Ring contraction, (5) N 6-ring substitution (reverse), (10) 6-ring annelation, (14) 2-Pyrone formation, (15) Dianhydride formation (incl. reverse), (21,19,20) N/O/S- 5-ring CH_2 substitution. Most commonly the molecule cannot be correctly represented in a Kekulé form, hinting to missing explicit hydrogens or radical structures, or potentially leading to the generation of non-unique SMILES identifiers. To avoid over-engineering of morphing operations, while maintaining a chemical space of formally valid molecules, detected errors in RDKit[1] molecular sanitation lead to removal of the molecule. Note, all cheminformatics-related tasks were carried out using RDKit, called from python 3.7, if not otherwise stated.

[a)] Electronic mail: reuter@fhi-berlin.mpg.de

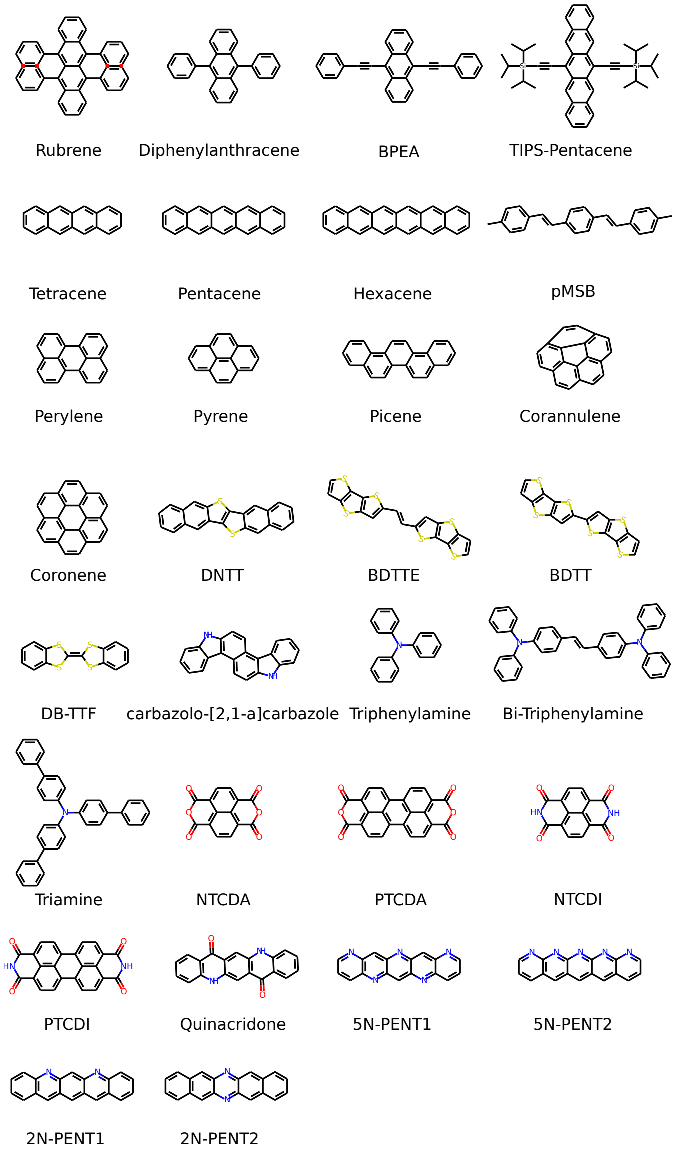

Supplementary Figure 1: Set of typical OSC-materials, collected for this work. Morphing operators for library enumeration were derived from this set.

Operations (1)-(6)

Ring contraction
[c:0]1[ch:1][ch][c:3][c!r5:4][c!r5:5]1>>[c:0]1[CH2:1][c:3][c:4][c:5]1

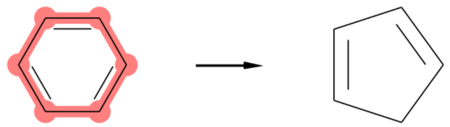

reverse Ring contraction
[CH2:0]1[c,C:1]-,=,:[c,C:2]-,=,:[c,C:3]-,=,:[c,C:4]1>>[ch:0]1c[c:1][c:2][c:3][c:4]1

Biphenyl addition
[cH&r6,CH&r5:1]>>[#6:1](-[c]1[ch]ccc[ch]1)

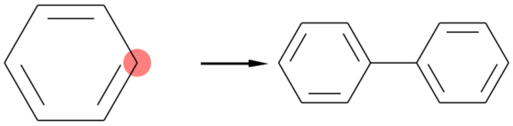

reverse Biphenyl addition
[c&r6,C&r5:1](-[c]1[ch]ccc[ch]1)>>[*:1]

Linkage doublebond
[ch&r6,CH1&r5:1]>>[*:1](-C=C-c1ccccc1)

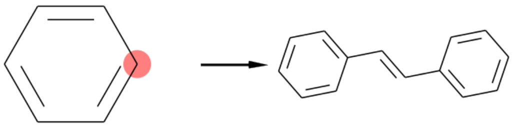

reverse Linkage doublebond
[c&r6,C&r5:1](-[C!R]=[C!R]-c1ccccc1)>>[ch&r6,CH1&r5:1]

Linkage triplebond
[ch&r6,CH1&r5:1]>>[*:1](-C#C-c1ccccc1)

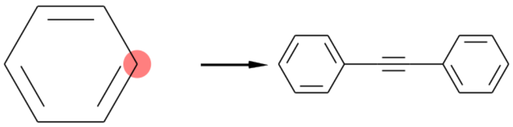

reverse Linkage triplebond
[c&r6,C&r5:1](-C#C-c1ccccc1)>>[ch&r6,CH1&r5:1]

N 6-ring substitution
[ch&r6:0]>>[nr6:0]

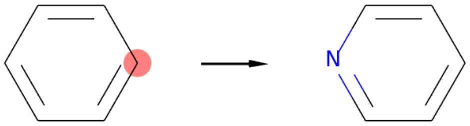

reverse N 6-ring substitution
[nr6:0]>>[ch&r6:0]

4-pyron formation
[ch:1]1[c:2][c:3][ch:4][c:5][c:6]1>>[o:1]1[c:2][c:3][C:4](=O)[c:5][c:6]1

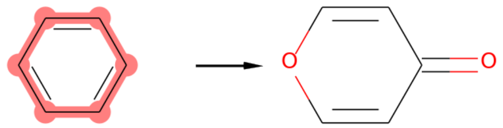

reverse 4-pyron formation
[o:1][c:2][c:3][c:4](=O)>>[ch:1][c:2][c:3][ch:4]

Operations (7)-(12)

O-N exchange
[o:1]>>[nH:1]

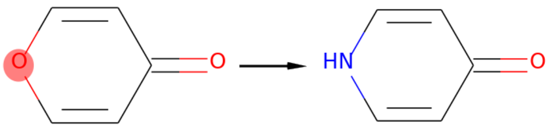

reverse O-N exchange
[nH:1]>>[o:1]

Phenylamine linkage
[ch&r6,CH1&r5:1]>>[*:1](-N-c1ccccc1)

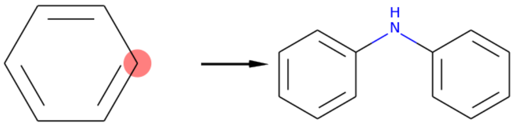

reverse Phenylamine linkage
[c&r6,C&r5:1](-[N!R]-c1ccccc1)>>[*:1]

Triphenylamine linkage
[r:1][NH!R:2][r:3]>>[r:1][N:2](-c1ccccc1)[r:3]

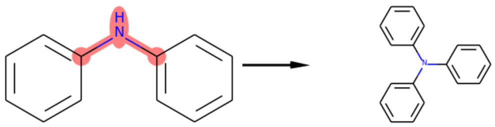

reverse Triphenylamine linkage
[r:1][N!R:2](-c1ccccc1)[r:3]>>[r:1][NH:2][r:3]

6-ring annelation
[cH&r6:1][cH&r6:2]>>[c:1]2cccc[c:2]2

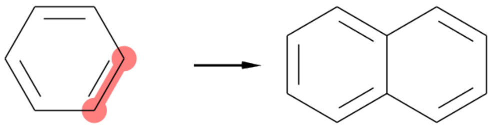

reverse 6-ring annelation
[r6:0][c:1]2[ch][ch][ch][ch][c:2]2[r6:3]>>[*:0][c:1][c:2][*:3]

5-ring annelation
[ch:1]2[c:3][c:4][c:5][c:6]3[c:7][c:8][c:9][ch:2][c:10]23>>C1=C[c:1]2[c:3][c:4][c:5][c:6]3[c:7][c:8][c:9][c:2]1[c:10]23

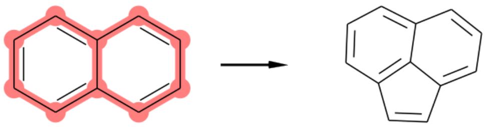

reverse 5-ring annelation
[ch,CH]1=,:[ch,CH][c:1]2[c:3][c:4][c:5][c:6]3[c:7][c:8][c:9][c:2]1[c:10]23>>[ch:1]2[c:3][c:4][c:5][c:6]3[c:7][c:8][c:9][ch:2][c:10]23

6-ring annelation 2
[c:4]1[c:3][ch:2][c:1]2[c:6]([c:5]1)[c:7][c:8][c:9]1[c:10][c:11][c:12][ch:13][c:14]12>>c1c[c:2]2[c:3][c:4][c:5][c:6]3[c:7][c:8][c:9]4[c:10][c:11][c:12][c:13]1[c:14]4[c:1]32

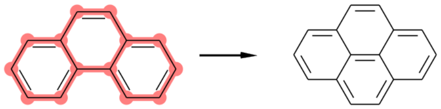

reverse 6-ring annelation 2
c1c[c:2]2[c:3][c:4][c:5][c:6]3[c:7][c:8][c:9]4[c:10][c:11][c:12][c:13]1[c:14]4[c:1]32>>[c:4]1[c:3][ch:2][c:1]2[c:6]([c:5]1)[c:7][c:8][c:9]1[c:10][c:11][c:12][ch:13][c:14]12

Supplementary Figure 2: List of morphing operations.

Operations (13)-(18)

6-ring annelation 3
[c:4]1[c:3][ch:2][c:1]2[c:6]([c:5]1)[c,C:7][c:9]1[c:10][c:11][c:12][ch:13][c:14]12>>c1c[c:2]2[c:3][c:4][c:5][c:6]3[*:7][c:9]4[c:10][c:11][c:12][c:13]1[c:14]4[c:1]3

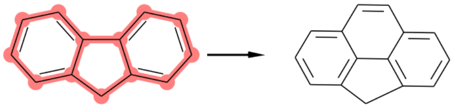

reverse 6-ring annelation 3
c1c[c:2]2[c:3][c:4][c:5][c:6]3[*:7][c:9]4[c:10][c:11][c:12][c:13]1[c:14]4[c:1]32>>[c:4]1[c:3][ch:2][c:1]2[c:6]([c:5]1)[*:7][c:9]1[c:10][c:11][c:12][ch:13][c:14]12

2-Pyrone formation
[c:1]1[c:2][ch:3][ch:4][c:5][c:6]1>>[c:1]1[c:2][o:3][c:4](=O)[c:5][c:6]1

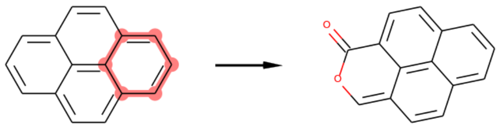

reverse 2-Pyrone formation
[o:1][c:2](=O)>>[ch:1][ch:2]

Dianhydride formation
[o:1][ch:2]>>[o:1][c:2](=O)

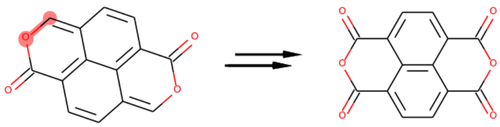

reverse Dianhydride formation
[o:1][c:2](=O)>>[o:1][ch:2]

Fulvalene formation
[r5:1][CH2r5:2][r5:3]>>[r5:1][C:2](=C1cccc1)[r5:3]

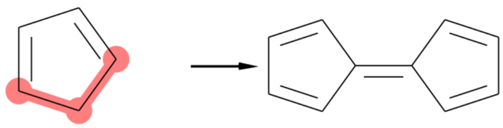

reverse Fulvalene formation
[r5:1][C:2](=C1cccc1)[r5:3]>>[r5:1][CH2r5:2][r5:3]

Dithiole formation
[CH,ch:0]1=[C,c:1][C,c:2]=[CH,ch:3][*:4]1>>[S:0]1[*:1]=,:[*:2][S:3][*:4]1

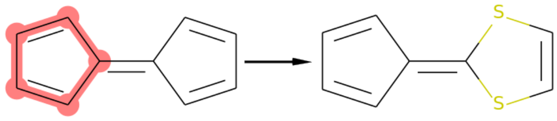

reverse Dithiole formation
[O,o,S,s:0]1[r5:1]-,=,:[r5:2][O,o,S,s:3][*:4]1>>[CH:0]1=[C:1][C:2]=[CH:3][*:4]1

Dioxole formation
[CH,ch:0]1=[C,c:1][C,c:2]=[CH,ch:3][*:4]1>>[O:0]1[*:1]=,:[*:2][O:3][*:4]1

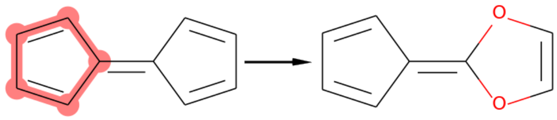

reverse Dioxole formation
[O,o,S,s:0]1[r5:1]-,=,:[r5:2][O,o,S,s:3][*:4]1>>[CH:0]1=[C:1][C:2]=[CH:3][*:4]1

Operations (19)-(22)

O 5-ring CH2 substitution
[#6H2r5:1]>>[O:1]

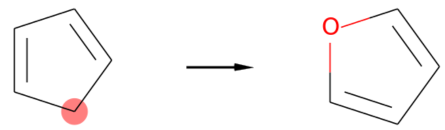

reverse O 5-ring CH2 substitution
[n,c:0]1[n,ch:1][o,O:2][n,c:3][n,c:4]1>>[*:0]1=[*:1][CH2:2][*:3]=[*:4]1

S 5-ring CH2 substitution
[#6H2r5:1]>>[S:1]

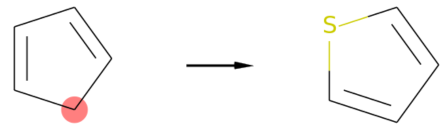

reverse S 5-ring CH2 substitution
[n,c:0]1[n,ch:1][s,S:2][n,c:3][n,c:4]1>>[*:0]1=[*:1][CH2:2][*:3]=[*:4]1

N 5-ring CH2 substitution
[#6H2r5:1]>>[NH:1]

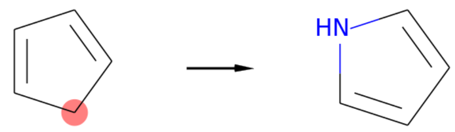

reverse N 5-ring CH2 substitution
[n,c:0]1[n,ch:1][NH:2][n,c:3][n,c:4]1>>[*:0]1=[*:1][CH2:2][*:3]=[*:4]1

N 5-ring CH substitution
[chr5,CH1r5:1]>>[n:1]

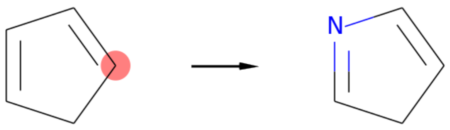

reverse N 5-ring CH substitution
[nr5:0]>>[ch&r5:0]

Supplementary Figure 2: Continued.

Supplementary Note 2: Methods

Symmetry detection: To focus on symmetric molecules —potentially more promising to be used as OSC materials—, the simple molecular morphing strategy was modified as shown in Figure 1 d) in the main text. In detail, full graph symmetry (1) was detected for a molecule if all heavy-atom environments occur at least two times. Environments are thereby compared by hashed subgraphs, extracted up to a bond radius of 5 around the central atom using the circular Morgan fingerprint machinery of RDKit. An asymmetric part of the molecule (2) can further by symmetrically substituted, and the molecule then still counts as symmetric. Central fragments that potentially allow for this type of symmetry to occur are bonded to more than one other fragment. Starting from these central fragments, the connections to all others are cleaved one by one, and the canonical SMILES strings[2] for the remaining part were recorded. If every resulting smiles string were found at least two times, the central fragment was symmetrically substituted according to our definition. In cases where the number of substitution sites on the central fragment was odd, substitution by an additional single fragment of at maximum 10 atoms was allowed without loosing the symmetry assignment. The size-limit was necessary to keep the symmetric part a large part of the molecule. Prosymmetry (3) was detected if at least one pair of similar carbon environments occurred and the central C atom was attached to at least one implicit H atom. Given that the tested molecule consisted of one core fragment the substituted molecule should then fall in class (1) or (2).

Enumeration of molecular test space: The test space of flexible π-conjugated OSC candidates was fully enumerated to benchmark the AML algorithm in a realistic setting. Enumeration started from benzene (generation 0). All morphing operations were then applied iteratively (generation by generation) to previously obtained full set of molecules. The so obtained offspring-molecules were kept when they were detected as being valid, symmetric and when satisfying molecular bounds as described in the main text. 65.552 unique molecules (identified by their canonical SMILES) were then exhaustively generated over the course of 14 generations, while no new molecules outside of the predefined bounds could be identified after, see Supplementary Figure 3 a).

Note also, that inclusion of reverse operations was found to increase inter-connectivity and the number of molecules that can overall be generated, as also shown in Supplementary Figure 3 a). Generated molecules were finally stored as canonical SMILES strings in a relational dataset containing parent-offspring information.

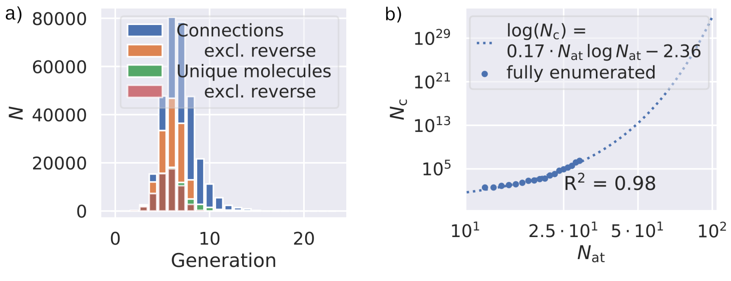

Supplementary Figure 3: a) Unique molecules and parent-offspring connections found in each fully enumerated generation. To probe the effect of reverse operations, the test-space used in this work is compared to a similar one enumerated without reverse operations. For the latter space, in total 55.771 unique molecules and 146.144 connections were found. Already after 10 generations no new molecules were found. b) Size-evolution of fully enumerated chemical spaces by number of compounds N_c as a function of the maximally allowed number of atoms N_{at}. R^2 applies for log-space.

Visualization of molecular test-space: The test space morphing network was visualized in a two-dimensional layout: An initial coordinate guess was obtained from Principal Components Analysis of the Graph[3] as implemented in the graphpca 0.5[4] python package. The final layout was then generated with the force-directed algorithm *Force-Atlas 2*[5], implemented in Gephi 0.9.2[6]. Gravity (of 40.0) and scaling (of 1.0) deviated from default values. Repulsion modification to prevent overlap was switched on in the final stages of layouting, spreading crowded parts over a larger area for better visibility. Note that these settings are given for completeness, affecting the final representation of the graph, but not the underlying morphing network.

Size estimation of virtually unlimited search space: The accessible number of compounds N_{c} in the virtually unlimited search space was estimated by extrapolation. Following Polishchuk et al.[7] we fitted the function $log(N_{\mathrm{c}}) = a \cdot N_{\mathrm{at}} \cdot log(N_{\mathrm{at}}) + b$ to reproduce the N_{c} progression of fully enumerated spaces bounded by a maximum number of atoms N_{at} in each molecule (including H), see Supplementary Figure 3 b). Given the imposed symmetry bounds and a finite set of morphing operations, the progression is flatter than originally reported by Polishchuk and co-workers, but does show a similar combinatorial explosion.

Descriptor calculation: For the calculation of electronic descriptors λ_{h} and ϵ_{HOMO}, initial 3D coordinates were generated from 2D molecular graphs using the default EKTDG[8] method implemented in RDKit. We relied on a protocol[9] implemented in the conformer generator deepchem 2.3.0[10]: While at maximum 5 rotatable bonds occur in the test-space (see below) we embedded a large number of 50 conformers for each molecular graph and initially relaxed them with the Merck Molecular Force Field (MMFF94)[11] as implemented in RDKit[12]. Note, for the production run, larger molecules are generated, and the number of embedded conformers was subsequently increased to the proposed maximum[9]. Duplicate geometries were then removed by a pruning step with an RMSD-threshold of 0.35 Å. The resulting conformers were kept, forming the initial conformer-ensemble of force-field geometries. The computationally efficient density functional tight-binding method – GFN1-xTB– of Grimme and co-workers[13,14] (v6.2.3) was then used for energy-based selection: All conformers were relaxed with the internal ANCOPT optimizer at the default geometry convergence criterion, selecting the lowest-energy structure and passing it to descriptor calculation.

Descriptors for the molecular test-space were then calculated for the lowest-energy vacuum geometry employing again the GFN1-xTB method. For λ_{h} the four-point scheme[15] was employed, while ϵ_{HOMO} was obtained for the neutral geometry. All GFN1-xTB results reported in the manuscript were then scaled to best fall in the range of DFT values obtained with the B3LYP[16–18] exchange-correlation functional. A comparison to DFT-B3LYP λ_{h} and ϵ_{HOMO} showed, that a reasonable scaling could be achieved by applying a linear correction to respective GFN1-xTB predictions, see Supplementary Figure 4 a) and b). As clearly not free from outliers, the linear fit was obtained by outlier-robust linear-regression using the RANSAC algorithm implemented in scikit-learn.[19,20] Deviating from default settings, residual thresholds of 50 meV and 0.3 eV were thereby chosen for λ_{h} and ϵ_{HOMO}, with at mininum 20 samples chosen randomly from original data. Note that B3LYP values for ϵ_{HOMO} were thereby obtained for the relaxed GFN1-xTB geometry.

In production runs, descriptor values were calculated for the lowest-energy vacuum geometry obtained from GFN1-xTB (v6.3.2), employing here the very tight geometry convergence criterion. For the large-molecules occuring in this setting, conformer-embedding could fail at default settings, and hence was restarted from random coordinates. The DFT-B3LYP level of theory was used as implemented in the FHI-aims code, employed with its established numeric atomic-orbital basis-sets and integration grid settings.[21,22] For λ_{h} computation, electronic wavefunctions were expanded in a tier 1 basis set and at light integration settings, employing for local optimizations the Broyden-Fletcher-

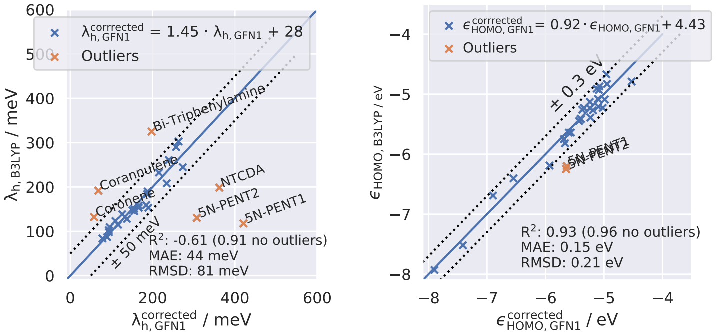

Supplementary Figure 4: Calibration of the GFN1-xTB method against DFT-B3LYP results of λ_h and ϵ_HOMO for 30 molecules contained in the set of typical OSC materials, see Supplementary Figure 1. The obtained linear fit is used to correct the raw GFN1-xTB results to the corrected values used throughout the present work. Outliers found by RANSAC linear regression are marked in orange.

Goldfarb-Shanno (BFGS) implemented in FHI-aims with a force convergence criterion $\mathrm{f}_\mathrm{max} < 0.025$ eV/Å. Dispersive forces were in the geometry relaxations were accounted for by the Tkatchenko-Schefer (TS)[23] method. We thereby slightly increased the aggregated energy tolerance to 10^{-3} eV, allowing small uphill steps during relaxation of flexible molecules. Based on this equilibrium structure, ϵ_HOMO predictions were obtained with an extended version of the tier 1 basis set[24] and light integration setting. Relativistic effects were treated on the level of the atomic zero-order regular approximation (atomic ZORA)[21].

Surrogate model: Gaussian Process Regression (GPR),[25,26] was used for surrogate modeling. A separate model was fitted for each descriptor ($d = \lambda_\mathrm{h}$ or ϵ_HOMO), using a training set $D = \{\mathrm{X}, \boldsymbol{y}_\mathrm{d}\}$ of the N labelled molecules contained in the current population. $\mathrm{X} = \{\boldsymbol{x}_1, ..., \boldsymbol{x}_N\}$ denote molecular descriptors (stored in vectors) and $\boldsymbol{y}_d = \{y_{d,1}, ..., y_{d,\mathrm{N}}\}$ descriptor values. F_acq is then calculated for the candidates of every learning step, using predictions and corresponding uncertainty estimates σ_{λ_h} and $\sigma_{\epsilon_\mathrm{HOMO}}$ obtained from the separately fitted models. Assuming λ_h and ϵ_HOMO to be uncorrelated, the necessary combined uncertainty σ is estimated by linear uncertainty propagation, as

$$\sigma^2 \approx \left\| \mathrm{F}^{-2} \cdot \mathbf{w}^4 \cdot \begin{pmatrix} \lambda_\mathrm{h} \cdot \sigma_{\lambda_\mathrm{h}} \\ \epsilon_\mathrm{align} \cdot \sigma_{\epsilon_\mathrm{HOMO}} \end{pmatrix}^2 \right\|_1 , \tag{1}$$

with dots denoting element-wise products.

In the employed GPR models value-prediction $f(\boldsymbol{x}')$ for a datapoint $\boldsymbol{x}'$ is not scalar, but follows the predictive Gaussian distribution

$$f_d(\boldsymbol{x}') \sim \mathcal{N}(\mu_d(\boldsymbol{x}'), \sigma_d^2(\boldsymbol{x}')) \tag{2}$$

This allows for simultaneous predictive uncertainty $\sigma_d(\boldsymbol{x}')$ estimation for the predicted property $\mu_d(\boldsymbol{x}')$, as

$$\mu_d(\boldsymbol{x}') = k(\boldsymbol{x}', X)[K + \sigma_n^2 I]^{-1}\boldsymbol{y}_d \tag{3}$$

$$\sigma_d^2(\boldsymbol{x}') = k(\boldsymbol{x}', \boldsymbol{x}') - k(\boldsymbol{x}', X)[K + \sigma_n^2 I]^{-1}k(X, \boldsymbol{x}') \tag{4}$$

Here, $k(\boldsymbol{x}, \boldsymbol{x}')$ is the covariance- or kernel function, measuring the similarity between $\boldsymbol{x}$ and $\boldsymbol{x}'$. K thereby is defined by $K_{i,j} = k(X_i, X_j)$ and known as the covariance or kernel matrix of the training set. Morgan fingerprints[27] as implemented in RDKit were used to generate descriptors $\boldsymbol{x}$, encoding

each molecule by the 2D molecular substructures present in it. Specifically, molecular substructures around each atom are extracted up to a bond radius of 2 and counted.

The covariance function is based on the MinMax kernel[28] k_d^m

$$k(\boldsymbol{x}, \boldsymbol{x}') = \sigma_v^2 \, k_d^m(\boldsymbol{x}, \boldsymbol{x}') \tag{5}$$

and closely related to the well-known Tanimoto kernel for molecular similarity computation on binary vectors[29]. While this choice, in contrast to other popular kernels does not feature adjustable hyperparameters fitted to the dataset, we found it to perform reasonably well for the prediction and uncertainty quantification, see below. The two model hyperparameters (noise level σ_n and vertical scale σ_v) are determined during model fitting, by log marginal likelihood maximization on the training-set[25]. Local optimization using L-BFGS as implemented in scipy is started 5 times with randomly (uniformly) sampled initial values, choosing the best final result. σ_n and σ_v were bounded between (0.001, 1.0) and (0.1, 3.0) respectively, and descriptor values $\boldsymbol{y}_d$ were scaled to zero mean and unit variance during internal use in the model. Our custom GPR implementation is based on respective code from scikit-learn.[20]

Supplementary Note 3: Intricacies of chemical space generation and descriptor calculation

A 2D molecular graph-based exploration of the huge chemical space of small organic molecules holds a number of potential pitfalls such as a potentially incomplete treatment of conformers or tautomeric forms.

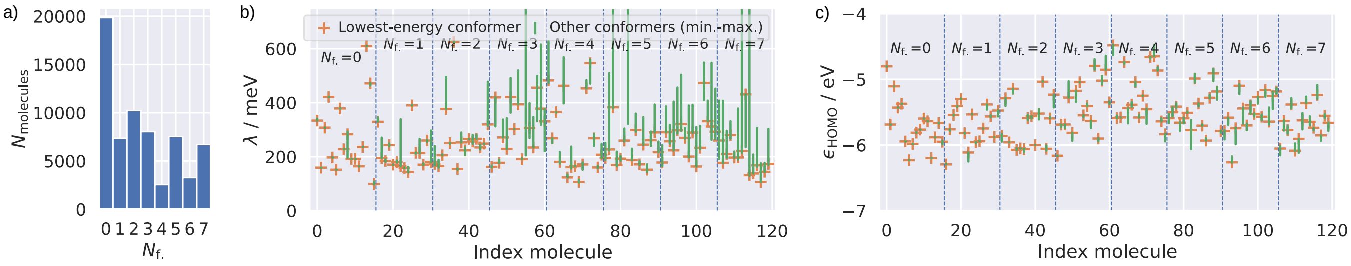

Supplementary Figure 5: a) Number of flexible bonds $N_{\text{f.}}$ found for molecules in the test-space. b) Variance in descriptor values for 120 molecules selected randomly from the molecular test-space, computed at the GFN1-xTB level of theory (corrected).

Confomers and tautomers: In a strict sense, descriptor values for λ_{h} and ϵ_{HOMO} are related to a specific conformer, as relative spatial orientations of molecular parts influence these electronic properties. Descriptors were here evaluated for the lowest-energy conformer identified in vacuum, and we correspondingly assume that the molecular graph encodes this structure. This approach should allow for a good first assessment of the studied molecular properties, with the caveat that the predominant solid-state conformation can be influenced by packing effects[30–32]. For the molecular test-space, a first assessment of conformer-related variance in λ_{h} and ϵ_{HOMO} occurring over the initial conformer-ensembles is here provided. At maximum 5 rotatable bonds occur in the test-space molecules, defined as single bonds not contained in a ring. Additional conformational flexibility can arise from stereocenters, that we did not specifically assign during morphing. Stereoisomers can however be generated during conformer search and the outcome is decided by energetic ranking. Stereocentres on tetrahedral (sp^3) carbon atoms are not incorporated during morphing, one (two) exocyclic double-bonds are however present in 7.318 (9.987) molecules, each potentially giving rise to E-/Z-Isomerism. A number of flexible bonds $N_{\text{f.}}$ is therefore here defined as the sum of occuring rotatable and exocyclic double-bonds in the molecule (see distribution in Supplementary Figure 5 a). At maximum 7 flexible bonds occur in the test-space molecules, and we randomly tested variance in λ_{h} and ϵ_{HOMO} for 15 molecules of each bin (0 to 7), see Supplementary Figure 5 b) and c). As expected, larger numbers of flexible bonds lead to larger spreads in descriptor values, but λ_{h} values for lowest-energy conformers quite favorably seem to provide a lower bound. A similar rule of thumb cannot be directly found for ϵ_{HOMO}, but the magnitudes of variances allow for a realistic estimate based on the lowest-energy conformer.

Finally, in our approach tautomers of the same molecule are recognized as separate entities owing to the uniqueness of the employed canonical SMILES strings. Given that the stability of each tautomer depends on the experimental conditions, a detailed exploration of them is far beyond the scope of this work.

Outliers after descriptor calculation: To check that the chemical integrity in the majority of molecules is preserved after the GFN1-xTB relaxation, we verified that bonding topologies still match with molecular graphs. We therefore read molecular SMILES and xyz-coordinate information in Open Babel[33,34] and compare resulting SMILES them after changing bond-orders to 1, and removing aromatic labels. Thereby, mismatches that could arise from differing assignments made due to varying interatomic distances in the xyz are circumvented[30,35]. We only found a negligible number of 16 molecules, in which this consistency check failed. The same test was carried out for relaxed structures of the charged molecular state, obtained during λ_{h} calculation with 599 structures failing the test. The latter usually manifests in high λ_{h} , regularly above 1000 meV. A second type of outlier could be identified, with 8 molecules showing a negative λ_{h}. There, neutral state geometry relaxation seems to have ended in a local minimum and restarting local relaxation from the charged state geometry lead to a different neutral state and positive λ_{h}. Due to the overall small number of outliers (< 1 %), and since this reflects a realistic situation in an unknown discovery task, we treated them as regular datapoints, with the added benefit of not disrupting the exhaustively enumerated morphing operation based network.

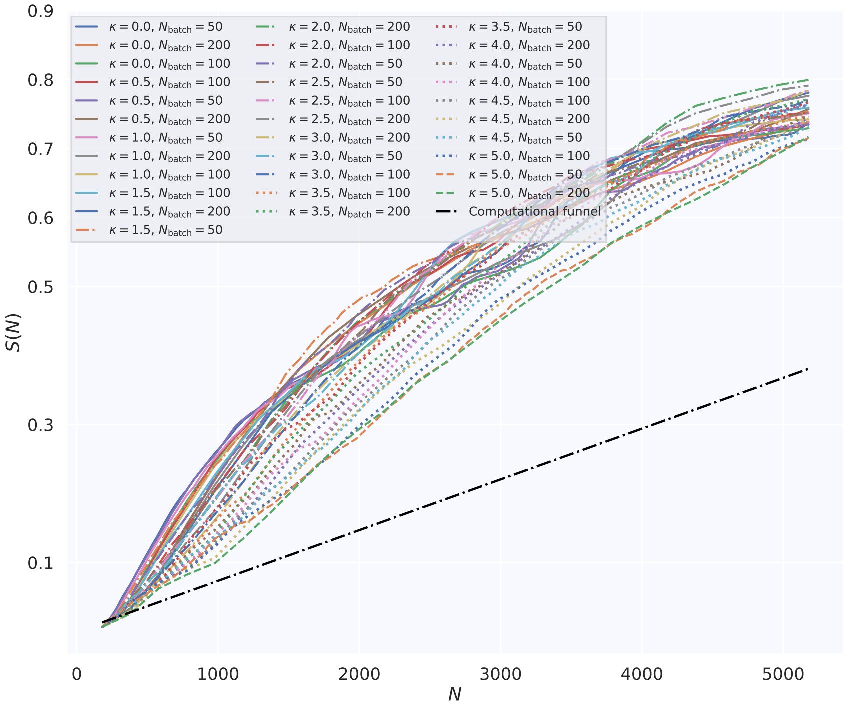

Supplementary Figure 6: Performance evaluation of AML-discovery, carried out at different hyperparameters. Candidates have thereby been generated by a one-fold application of all morphing operations to the current population. A twodimensional grid in the ranges of $\kappa = 0.0$, 0.5, 1.0, 1.5, 2.0, 2.5, 3.0, 4.0 and 5.0, and of $N_{\text{batch}} = 50$, 100 and 200 was evaluated.

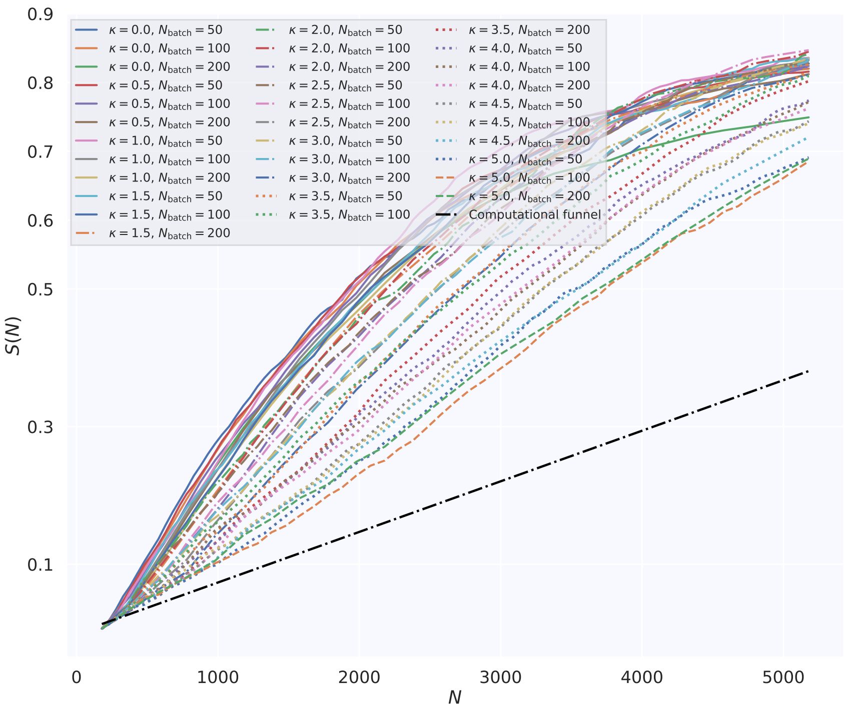

Supplementary Figure 7: See Supplementary Figure 6 for description. Candidates have thereby been generated by a two-fold application of all morphing operations to the current population.

Supplementary Table I: AML discovery success S(5179) for size-restricted candidate sets, determined by N_{deep} and d_{search}, see text. Each cell provides the median values of S(5179) obtained over 5 runs, while a maximum deviation of only ± 0.06 was found between runs and separate sampling errors are not stated. For comparison note again that S(5179) values of 0.78 and 0.85 are found upon one-fold/two-fold full application of all morphing operations. The setting marked in bold letters was finally used for exploration in a virtually unlimited space

N_{deep}	100	250	500	1000
$d_{\text{search}} = 1$	0.50	0.64	0.70	0.72
$d_{\text{search}} = 2$	0.63	0.72	0.78	0.80
$d_{\text{search}} = 3$	0.68	0.78	**0.82**	0.84
$d_{\text{search}} = 4$	0.71	0.77	0.82	0.83
$d_{\text{search}} = 5$	0.70	0.79	0.82	0.83
$d_{\text{search}} = 10$	0.73	0.81	0.81	0.83

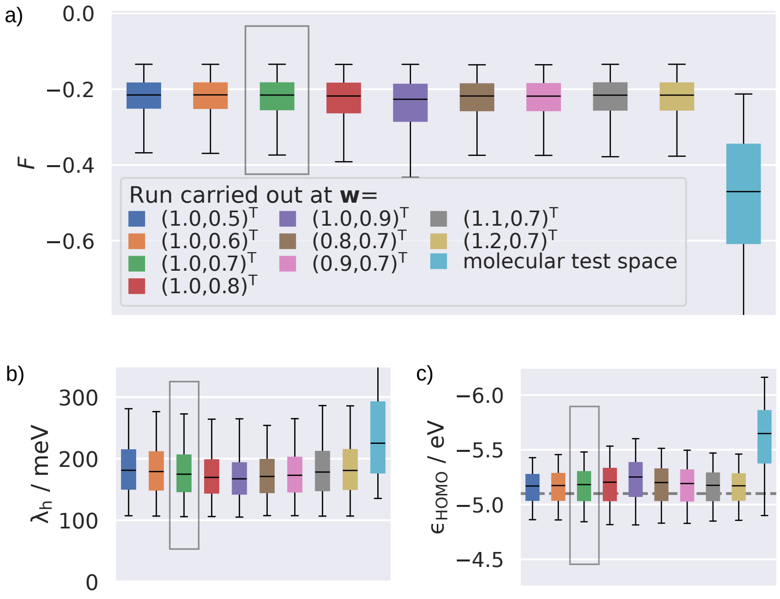

Supplementary Figure 8: Sensitivity analysis of AML discovery success with respect to the specific weight vector **w** (equation 1), shown as boxplots of the respective distributions. The interquartile range (IQR) of the data is shown by the boxes, while black lines in them denote the distribution-medians. Whiskers (vertical lines extending beyond boxes) show the spread of the data extending to the 5 % and 95 % percentiles. As only the overall shape of the distributions is here relevant, data points beyond these limits have been omitted from the visualization. Within the range of studied variation, the discovery success remained stable and was biased to the useful region of descriptor values. The analysis was carried out in the exhaustive molecular test space for the intermediate value pair of $(N_{\mathrm{batch}}, \kappa) = (100, 2.5)$ and onefold application of all morphing operations as described in the text. Note to allow for direct comparison, F values displayed in a) were calculated using $\mathbf{w} = (1.0, 0.7)^{\top}$ as stated in the text. The respective default run applying these weights during discovery is highlighted by frames. A background distribution for the molecular test space is shown as the rightmost boxplot.

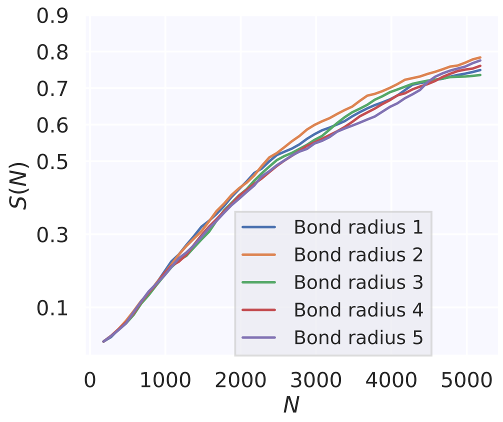

Supplementary Figure 9: Sensitivity analysis of AML discovery success with respect to the bond radius applied in the circular Morgan fingerprints. The analysis was carried out in the exhaustive molecular test space for the intermediate value pair of $(N_{\mathrm{batch}}, \kappa) = (100, 2.5)$ and onefold application of all morphing operations as described in the text.

Supplementary Note 4: First-principles AML discovery in a virtually unlimited OSC chemical space

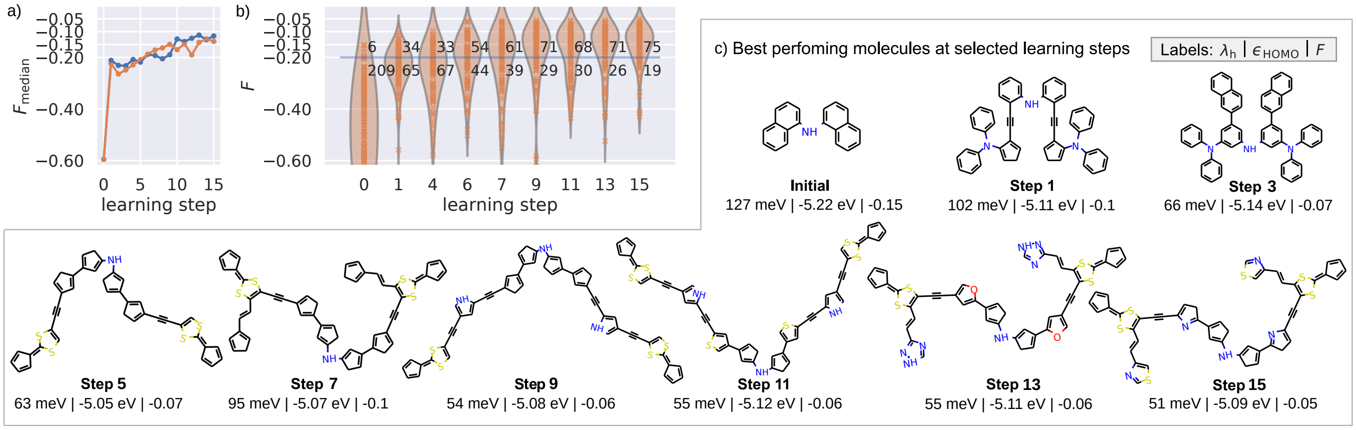

Supplementary Figure 10: First-principles AML discovery in a virtually unlimited space, re-executed to study its robustness. The layout thereby closely follows Fig. 4 of the main text and comments given therein apply. In a), results of the re-execution are shown as an orange trace, while the blue trace reproduces the results of the AML discovery run presented in the main text (Fig. 4).

To assess the robustness of AML discovery in the virtually unlimited OSC chemical space, we executed the algorithm a second time, applying similar settings. Supplementary Figure 10 a,b) presents the corresponding results in an analogous way as Fig. 4 in the main text does for the first AML discovery run. Overall, highly similar behavior and performance is found. After 15 learning steps, first-principles calculations for 1693 molecules had successfully finished, among them 840 molecules that surpassed a molecular fitness of $F \geq -0.2$. The relative success rate of 50 % was then only slightly lower than the 54 % described in the main text. Again, only for a minority of molecules (22) the descriptor calculations terminated unsuccessfully. We also note, that the re-execution fully relied on a first-principles calculation of all requested molecular descriptor values, here not drawing any information from the internal database of computed values that had accumulated during development and testing of the AML discovery algorithm. This assessment thus also eliminated any bias that might arise from selective presence of descriptor values in the database. Clear differences between both executions however arise in the uncovered favorable molecular structures, compare e.g. Supplementary Figure 10 c) to Figure 4 c). Different parts of the virtually unlimited chemical OSC space have correspondingly been explored, as random elements are inherent to our search space reduction strategy and also enter with descriptor calculations becoming available at different times on the HPC system.

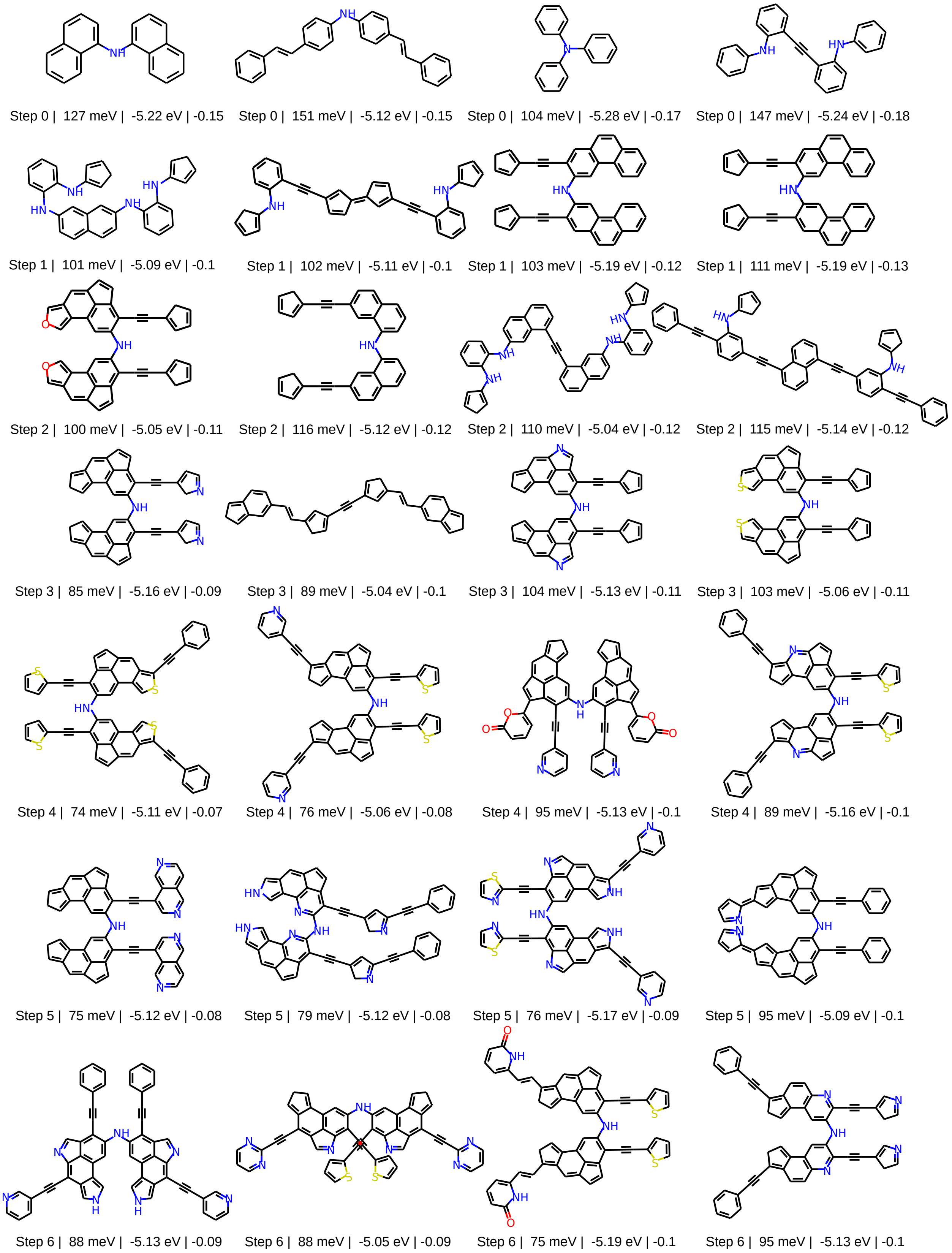

Supplementary Figure 11: Extended list showing the 4 best performing molecules identified at each learning step during the AML discovery in a virtually unlimited space and at the DFT-B3LYP (+vdW) level of theory. Note, overlaps are marked in red. Continued on next page.

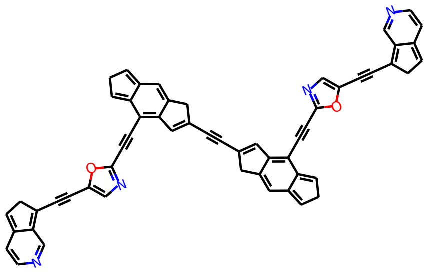

Step 7 | 109 meV | -5.08 eV | -0.11

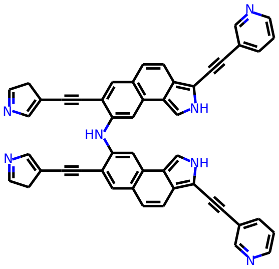

Step 7 | 83 meV | -5.09 eV | -0.08

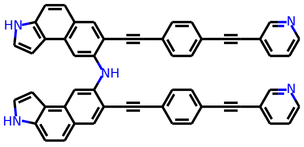

Step 7 | 106 meV | -5.06 eV | -0.11

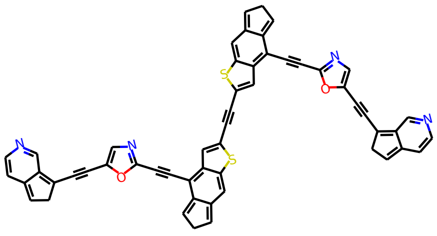

Step 7 | 104 meV | -5.04 eV | -0.11

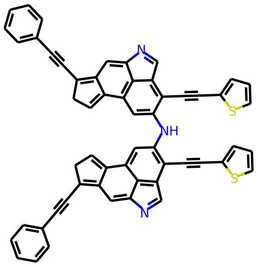

Step 8 | 82 meV | -5.12 eV | -0.08

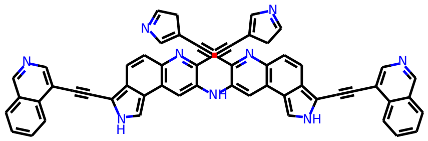

Step 8 | 82 meV | -5.21 eV | -0.11

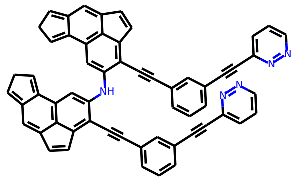

Step 8 | 102 meV | -5.1 eV | -0.1

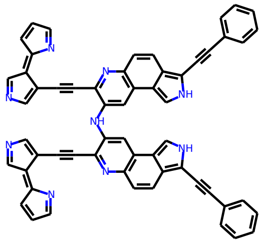

Step 8 | 108 meV | -5.15 eV | -0.11

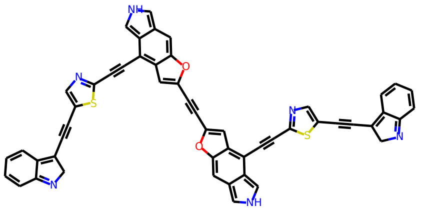

Step 9 | 76 meV | -5.09 eV | -0.08

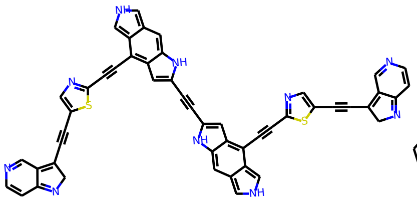

Step 9 | 58 meV | -5.03 eV | -0.08

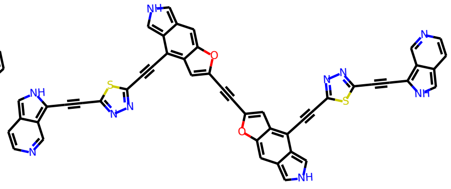

Step 9 | 95 meV | -5.1 eV | -0.1

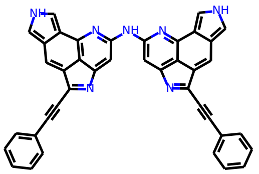

Step 9 | 102 meV | -5.1 eV | -0.1

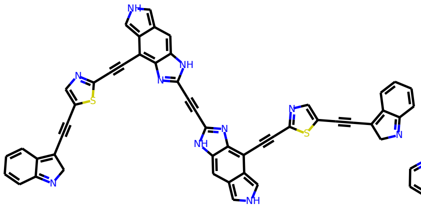

Step 10 | 66 meV | -5.09 eV | -0.07

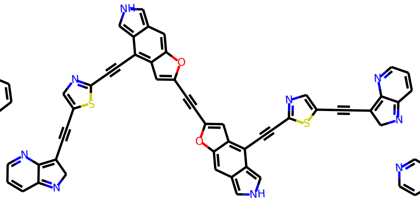

Step 10 | 74 meV | -5.1 eV | -0.07

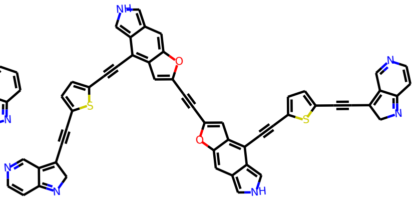

Step 10 | 73 meV | -5.13 eV | -0.08

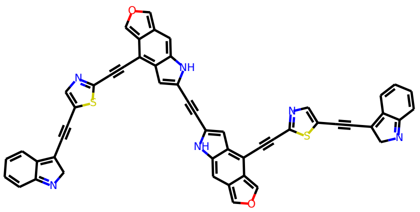

Step 10 | 74 meV | -5.12 eV | -0.08

Supplementary Figure 11: Continued.

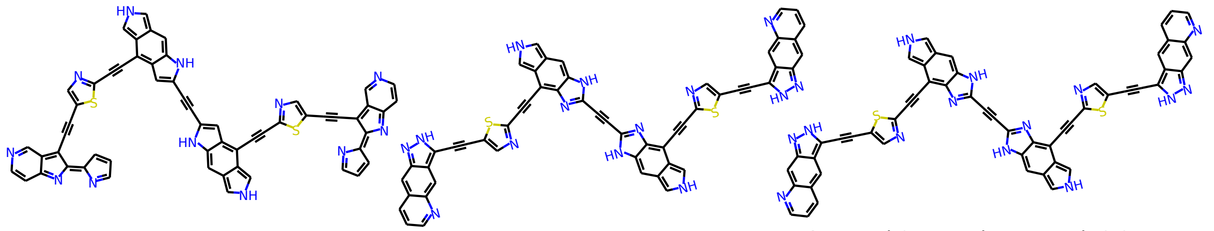

Step 11 | 54 meV | -5.16 eV | -0.07   Step 11 | 67 meV | -5.08 eV | -0.07   Step 11 | 67 meV | -5.11 eV | -0.07

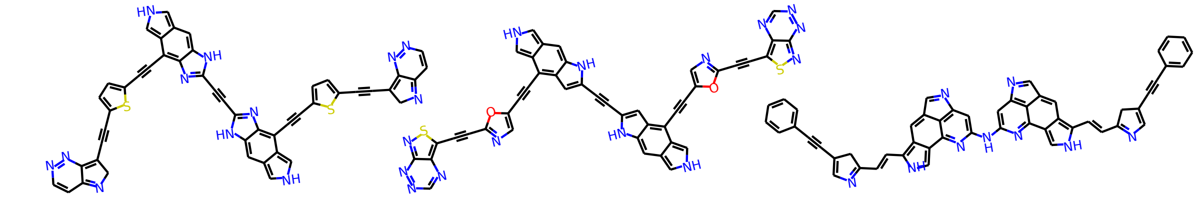

Step 11 | 57 meV | -5.16 eV | -0.07   Step 12 | 57 meV | -5.08 eV | -0.06   Step 12 | 59 meV | -5.1 eV | -0.06

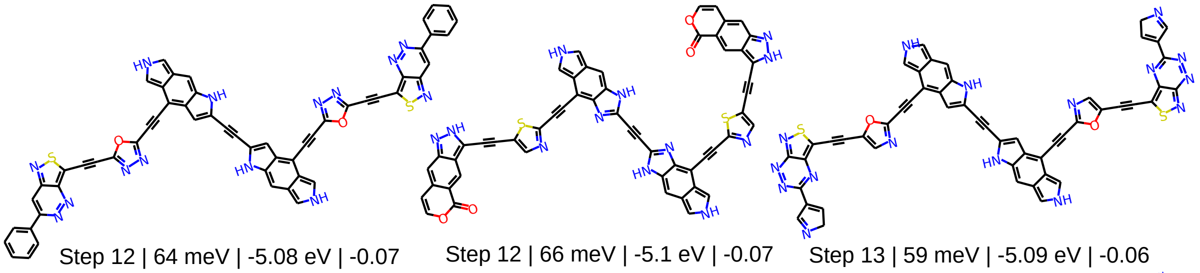

Step 12 | 64 meV | -5.08 eV | -0.07   Step 12 | 66 meV | -5.1 eV | -0.07   Step 13 | 59 meV | -5.09 eV | -0.06

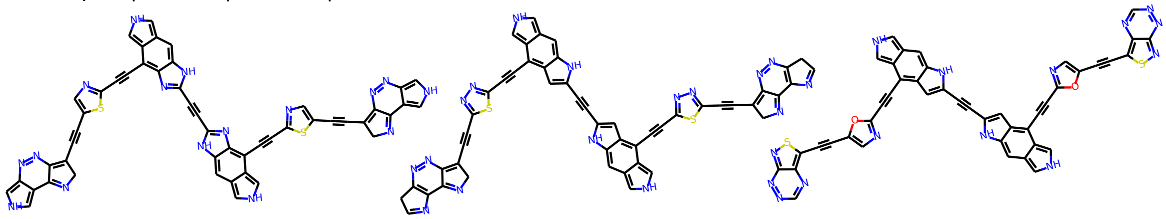

Step 13 | 62 meV | -5.09 eV | -0.06   Step 13 | 63 meV | -5.11 eV | -0.06   Step 13 | 59 meV | -5.14 eV | -0.06

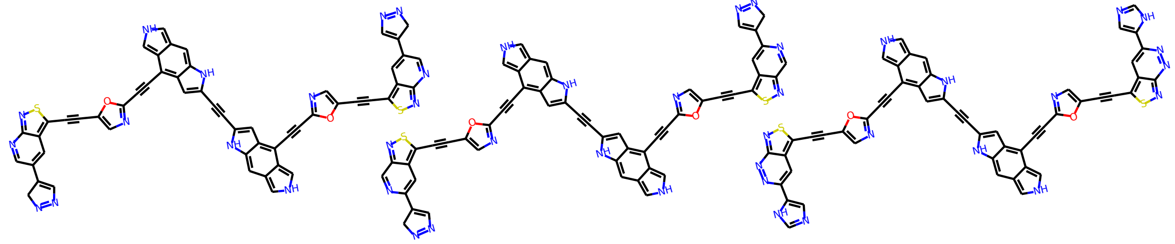

Step 14 | 57 meV | -5.12 eV | -0.06   Step 14 | 56 meV | -5.07 eV | -0.06   Step 14 | 59 meV | -5.08 eV | -0.06

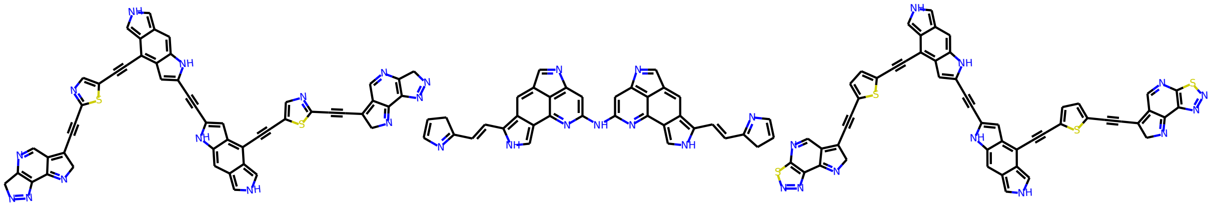

Step 14 | 56 meV | -5.13 eV | -0.06   Step 15 | 57 meV | -5.12 eV | -0.06   Step 15 | 59 meV | -5.08 eV | -0.06

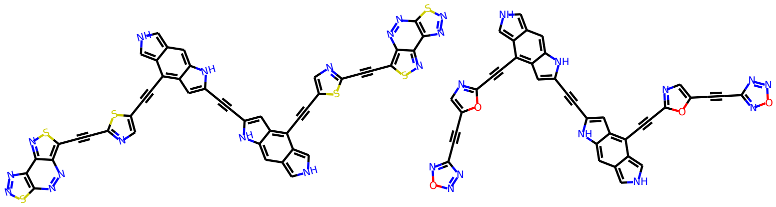

Step 15 | 61 meV | -5.12 eV | -0.06   Step 15 | 64 meV | -5.09 eV | -0.06

Supplementary Figure 11: Continued.

Supplementary References

[1] "The RDKit: Open-Source Cheminformatics Software, version 2019.09.3., 2019," `http://www.rdkit.org`.

[2] D. Weininger, A. Weininger, and J. L. Weininger, "Smiles. 2. algorithm for generation of unique smiles notation," J. Chem. Inf. Comput. Sci. **29**, 97–101 (1989).

[3] M. Saerens, F. Fouss, L. Yen, and P. Dupont, "The principal components analysis of a graph, and its relationships to spectral clustering," in *Machine Learning: ECML 2004*, edited by J.-F. Boulicaut, F. Esposito, F. Giannotti, and D. Pedreschi (Springer Berlin Heidelberg, Berlin, Heidelberg, 2004) pp. 371–383.

[4] B. Istenes, "graphpca, version 0.5," `https://github.com/brandones/graphpca` (2020).

[5] M. Jacomy, T. Venturini, S. Heymann, and M. Bastian, "Forceatlas2, a continuous graph layout algorithm for handy network visualization designed for the gephi software," PLOS ONE **9**, 1–12 (2014).

[6] M. Bastian, S. Heymann, and M. Jacomy, "Gephi: An open source software for exploring and manipulating networks," in *International AAAI Conference on Weblogs and Social Media* (2009).

[7] P. G. Polishchuk, T. I. Madzhidov, and A. Varnek, "Estimation of the size of drug-like chemical space based on gdb-17 data," J. Comput. Aided Mol. Des. **27**, 675–679 (2013).

[8] S. Riniker and G. A. Landrum, "Better informed distance geometry: Using what we know to improve conformation generation," J. Chem. Inf. Model. **55**, 2562–2574 (2015).

[9] J.-P. Ebejer, G. M. Morris, and C. M. Deane, "Freely available conformer generation methods: How good are they?" J. Chem. Inf. Model. **52**, 1146–1158 (2012).

[10] B. Ramsundar, P. Eastman, P. Walters, V. Pande, K. Leswing, and Z. Wu, *Deep Learning for the Life Sciences* (O'Reilly Media, 2019).

[11] T. A. Halgren, "Merck molecular force field. I. basis, form, scope, parameterization, and performance of mmff94," J. Comput. Chem. **17**, 490–519 (1996).

[12] P. Tosco, N. Stiefl, and G. Landrum, "Bringing the mmff force field to the rdkit: implementation and validation," J. Cheminformatics **6**, 37 (2014).

[13] S. Grimme, C. Bannwarth, and P. Shushkov, "A robust and accurate tight-binding quantum chemical method for structures, vibrational frequencies, and noncovalent interactions of large molecular systems parametrized for all spd-block elements (z = 186)," J. Chem. Theory Comput. **13**, 1989–2009 (2017).

[14] "grimme-lab/xtb: Semiempirical extended tight-binding program package," `https://github.com/grimme-lab/xtb` (2020).

[15] S. F. Nelsen, S. C. Blackstock, and Y. Kim, "Estimation of inner shell marcus terms for amino nitrogen compounds by molecular orbital calculations," J. Am. Chem. Soc. **109**, 677–682 (1987).

[16] A. D. Becke, "Density-functional exchange-energy approximation with correct asymptotic behavior," Phys. Rev. A **38**, 3098–3100 (1988).

[17] C. Lee, W. Yang, and R. G. Parr, "Development of the colle-salvetti correlation-energy formula into a functional of the electron density," Phys. Rev. B **37**, 785–789 (1988).

[18] P. J. Stephens, F. J. Devlin, C. F. Chabalowski, and M. J. Frisch, "Ab initio calculation of vibrational absorption and circular dichroism spectra using density functional force fields," J. Phys. Chem. **98**, 11623–11627 (1994).

[19] F. Pedregosa, G. Varoquaux, A. Gramfort, V. Michel, B. Thirion, O. Grisel, M. Blondel, P. Prettenhofer, R. Weiss, V. Dubourg, *et al.*, "Scikit-learn: Machine learning in python," J. Mach. Learn. Res. **12**, 2825–2830 (2011).

[20] "scikit-learn: machine learning in python, version 0.22.1," `https://github.com/scikit-learn/scikit-learn` (2020).

[21] V. Blum, R. Gehrke, F. Hanke, P. Havu, V. Havu, X. Ren, K. Reuter, and M. Scheffler, "Ab initio molecular simulations with numeric atom-centered orbitals," Comp. Phys. Commun. **180**, 2175–2196 (2009).

[22] I. Y. Zhang, X. Ren, P. Rinke, V. Blum, and M. Scheffler, "Numeric atom-centered-orbital basis sets with valence-correlation consistency from h to ar," New J. Phys. **15**, 123033 (2013).

[23] A. Tkatchenko and M. Scheffler, "Accurate molecular van der Waals interactions from ground-state electron density and free-atom reference data," Phys. Rev. Lett. **102**, 073005 (2009).

[24] C. Schober, K. Reuter, and H. Oberhofer, "Critical analysis of fragment-orbital dft schemes for the calculation of electronic coupling values," J. Chem. Phys. **144**, 054103 (2016).

[25] C. Rasmussen and C. Williams, *Gaussian Processes for Machine Learning*, Adaptative computation and machine learning series (University Press Group Limited, 2006).

[26] C. Williams and C. Rasmussen, "Gaussian processes for regression," in *Advances in neural information processing systems*, Max-Planck-Gesellschaft (MIT Press, Cambridge, MA, USA, 1996) pp. 514–520.

[27] D. Rogers and M. Hahn, "Extended-connectivity fingerprints," J. Chem. Inf. Model. **50**, 742–754 (2010).

[28] L. Ralaivola, S. J. Swamidass, H. Saigo, and P. Baldi, "Graph kernels for chemical informatics," Neural Netw. **18**, 1093 – 1110 (2005).

[29] D. Bajusz, A. Rácz, and K. Héberger, "Why is tanimoto index an appropriate choice for fingerprint-based similarity calculations?" J. Cheminformatics **7**, 20 (2015).

[30] A. Stuke, C. Kunkel, D. Golze, M. Todorovic, J. T. Margraf, K. Reuter, P. Rinke, and H. Oberhofer, "Atomic structures and orbital energies of 61,489 crystal-forming organic molecules," Sci. Data **7**, 58 (2020).

[31] N. W. Mitzel and D. W. H. Rankin, "Saracen molecular structures from theory and experiment: the best of both worlds," Dalton Trans. , 3650–3662 (2003).

[32] S. Blomeyer, M. Linnemannstoens, J. H. Nissen, J. Paulus, B. Neumann, H.-G. Stammler, and N. W. Mitzel, "Intramolecular interactions in flexibly linked partially fluorinated bisarenes in the gas phase," Angew. Chem. Int. Ed. **56**, 13259–13263 (2017).

[33] N. M. O'Boyle, M. Banck, C. A. James, C. Morley, T. Vandermeersch, and G. R. Hutchison, "Open babel: An open chemical toolbox," J. Cheminformatics **3**, 33 (2011).

[34] "Open Babel, version 3.1.0," `https://github.com/openbabel/openbabel` (2020), [Online; accessed 10-May-2020].

[35] R. Ramakrishnan, P. O. Dral, M. Rupp, and O. A. von Lilienfeld, "Quantum chemistry structures and properties of 134 kilo molecules," Sci. Data **1** (2014).
