# Supplementary material for: Active discovery of organic semiconductors
Source: Nat Commun. 2021 Apr 23;12:2422. doi: 10.1038/s41467-021-22611-4 (PMC8065160; doi:10.1038/s41467-021-22611-4)
Supplement: Supplementary file 2 — Description of Additional Supplementary Files [file 41467_2021_22611_MOESM2_ESM.docx]

**Description of Additional Supplementary Files**

File Name: Supplementary Movie 1

Description: Trajectory accompanying Figure 3(upper panel)

File Name: Supplementary Movie 2

Description: Trajectory accompanying Figure 3(middle panel)

File Name: Supplementary Movie 3

Description: Trajectory accompanying Figure 3(lower panel)
